# The ER membrane-anchored ubiquitin ligase Hrd1 is a positive regulator of T-cell immunity

**DOI:** 10.1038/ncomms12073

**Published:** 2016-07-15

**Authors:** Yuanming Xu, Fang Zhao, Quan Qiu, Kun Chen, Juncheng Wei, Qingfei Kong, Beixue Gao, Johanna Melo-Cardenas, Bin Zhang, Jinping Zhang, Jianxun Song, Donna D. Zhang, Jianing Zhang, Yunping Fan, Huabin Li, Deyu Fang

**Affiliations:** 1 Department of Pathology, Northwestern University Feinberg School of Medicine, 303 E Chicago Avenue, Chicago, Illinois 60611, USA; 2 Allergy Center, Department of Otolaryngology-Head and Neck Surgery, Affiliated Eye and ENT Hospital, Fudan University, Shanghai 200031, China; 3 Robert H. Lurie Comprehensive Cancer Center, Department of Medicine-Division of Hematology/Oncology, Northwestern University Feinberg School of Medicine, Chicago, Illinois 60611, USA; 4 Institutes of Biology and Medical Sciences, Soochow University, Suzhou, Jiangsu Province 215123, China; 5 Department of Microbiology and Immunology, The Pennsylvania State University College of Medicine, Hershey, Pennsylvania 17033, USA; 6 Department of Pharmacology and Toxicology, College of Pharmacy, University of Arizona, Tucson, Arizon 85721, USA; 7 Department of Biochemistry, School of Life Science and Medicine, Dalian University of Technology, 2 Linggong Road, Dalian 116024, China; 8 Guangdong Provincial Engineering Research Center for Molecular Imaging, The Fifth Affiliated Hospital, Sun Yat-sen University, Zhuhai 519000, China

## Abstract

Identification of positive regulators of T-cell immunity induced during autoimmune diseases is critical for developing novel therapies. The endoplasmic reticulum resident ubiquitin ligase Hrd1 has recently emerged as a critical regulator of dendritic cell antigen presentation, but its role in T-cell immunity is unknown. Here we show that genetic deletion of Hrd1 in mice inhibits T-cell proliferation, production of IL-2, and differentiation of Th1 and Th17 cells, and consequently protects mice from experimental autoimmune encephalomyelitis. Hrd1 facilitates T-cell proliferation by the destruction of cyclin-dependent kinase inhibitor p27^kip1^, and deletion of p27^kip1^ in Hrd1-null T-cells rescues proliferative capacity but not the production of cytokines, including IL-2, IFN-γ and IL-17. T-cell expression of Hrd1 is higher in patients with multiple sclerosis than in healthy individuals, and knockdown of Hrd1 in human CD4^+^ T cells inhibits activation and differentiation to Th1 and Th17 cells. Our study identifies Hrd1 as a previously unappreciated positive regulator of T cells and implies that Hrd1 is a potential therapeutic target for autoimmune diseases.

## Abstract (web summary)

Hrd1 is an E3 ubiquitin ligase involved in ER-associated degradation and MHC I turnover. Here the authors use T-cell-specific ko mice and a mouse model of multiple sclerosis to show that Hrd1 also drives pro-inflammatory T helper cell responses and contributes to pathogenesis of autoimmune disease.

## Graphical abstract

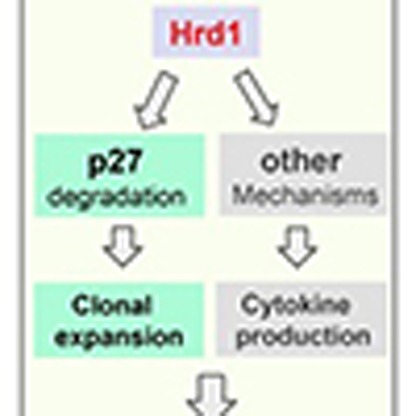

T-cell activation is initiated by the binding of antigenic peptides presented by the major histocompatibility complex (MHC) to the T-cell receptor (TCR)/CD3 complex, which results in T-cell proliferation and interleukin-2 (IL-2) production^1^,^2^. In addition to antigen-specific interaction with the TCR, full-scale T-cell activation requires a co-stimulatory signal provided by engagement of the T-cell co-receptor CD28 with its ligand, B7, on antigen-presenting cells^2^. Stimulation of TCR and CD28 drives T cells to proliferate by increasing the expression and activity of positive regulators and suppressing the expression of negative regulators through the activation of several transcription factors, including AP-1, NF-κB and NF-AT, and through epigenetic regulation^2^. For example, the expression of genes that promote cell cycle progression, including cyclins and cyclin-dependent kinases (CDKs), is quickly induced on TCR/CD28 stimulation, both *in vitro* and *in vivo*. By contrast, the expression of genes that inhibit cell cycle progression, including the CDK inhibitors and retinoblastoma protein, diminishes within a few hours of T-cell activation^3^,^4^. After clonal expansion, an efficient T-cell immune response relies on the differentiation of T cells into their functional subsets; CD4^+^ helper T (Th) cells differentiate into Th1, Th2, Th17 or regulatory T cells (Tregs). The differentiation programs resulting in these Th-cell subsets are influenced by a number of factors, including the dose and form of antigen and the antigen-presenting cells and/or co-stimulatory molecules; however, the dominant regulators of Th-cell differentiation are cytokines, often produced by innate immune cells such as dendritic cells. Commitment to each Th subset is regulated by a variety of mechanisms, including differential cytokine signalling, exclusive cytokine receptor expression, differential expression of transcription factors and/or differential chromatin remodelling of lineage-specific genes^5^. Although substantial progress has been made, the molecular mechanisms that govern T-cell activation and differentiation are incompletely understood.

Hrd1 is an E3 ubiquitin ligase that spans the endoplasmic reticulum (ER) membrane and was initially identified as a human homologue of yeast Hrd1p/Der3p (refs 6, 7). Hrd1 forms a complex with its stabilizing factor SEL1L, a human homologue of yeast Hrd3p (ref. 8). The best-characterized function of Hrd1 is to catalyse the degradation of misfolded/unfolded proteins, a process called ER-associated degradation (ERAD)^9^,^10^. MHC I heavy chain is an endogenous substrate for Hrd1, and Hrd1 suppression leads to the accumulation of glycosylated MHC I in the ER membrane^11^. ERAD-mediated MHC I degradation probably has an important role in the homeostatic regulation of MHC class I assembly and expression by dendritic cells^11^,^12^. The *Hrd1* gene has been renamed *SYVN1* (Synoviolin), owing to induced expression by synovial fibroblasts from patients with rheumatoid arthritis (RA), a disease in which Hrd1 suppresses synovial cell apoptosis^13^,^14^. We and others have demonstrated that pro-inflammatory cytokines, including IL-1β, IL-6, tumour necrosis factor-α (TNF-α) and IL-17, which have important pathogenic roles in synovitis development, induce Hrd1 expression in RA^15^,^16^,^17^. A body of evidence now indicates that Hrd1 also has a variety of important ERAD-independent physiological and pathological functions. p53 was the first identified non-ERAD substrate of Hrd1, and p53 ubiquitination and degradation negatively regulate Hrd1 expression and functions, including gene transcription, cell cycle regulation and apoptosis^18^. In addition to p53, the transcription factor Nrf2 is a substrate of Hrd1 in hepatocytes, with ubiquitination leading to attenuation of the Nrf2-mediated anti-oxidative stress response during liver cirrhosis^19^. Moreover, we have shown that Hrd1 programs dendritic cells for CD4^+^ T-cell activation during inflammation by directly targeting the zinc-finger transcription suppressor Blimp1 for ubiquitination and degradation. As Blimp1 suppresses the transcription of MHC class II, dendritic cell Hrd1 promotes CD4^+^ T-cell priming by inducing MCH II expression^20^.

In the current study, we conditionally delete the *Hrd1* gene in developing thymocytes by crossing floxed Hrd1 and CD4-Cre mice. By analysing the phenotype of the resulting T-cell-specific Hrd1 conditional knockout (cKO) mice, we show that Hrd1 functions are required for T-cell homeostasis, activation and differentiation. Targeted *Hrd1* gene deletion reduced T-cell numbers, inhibited T-cell clonal expansion and attenuated CD4^+^ T-cell differentiation to Th1, Th17 and Treg lineages. At the molecular level, we identify p27^Kip1^ as a target of the Hrd1 E3 ubiquitin ligase, as Hrd1 interacts with p27^kip1^ and promotes its degradation in T cells. Deletion of p27^kip1^ in Hrd1 cKO T cells rescues proliferation but not differentiation of T cells. Therefore, we identify Hrd1 as a positive regulator of T-cell immunity.

## Results

### Mice with T-cell-specific Hrd1 deletion are lymphocytopenic

**Figure 1:**
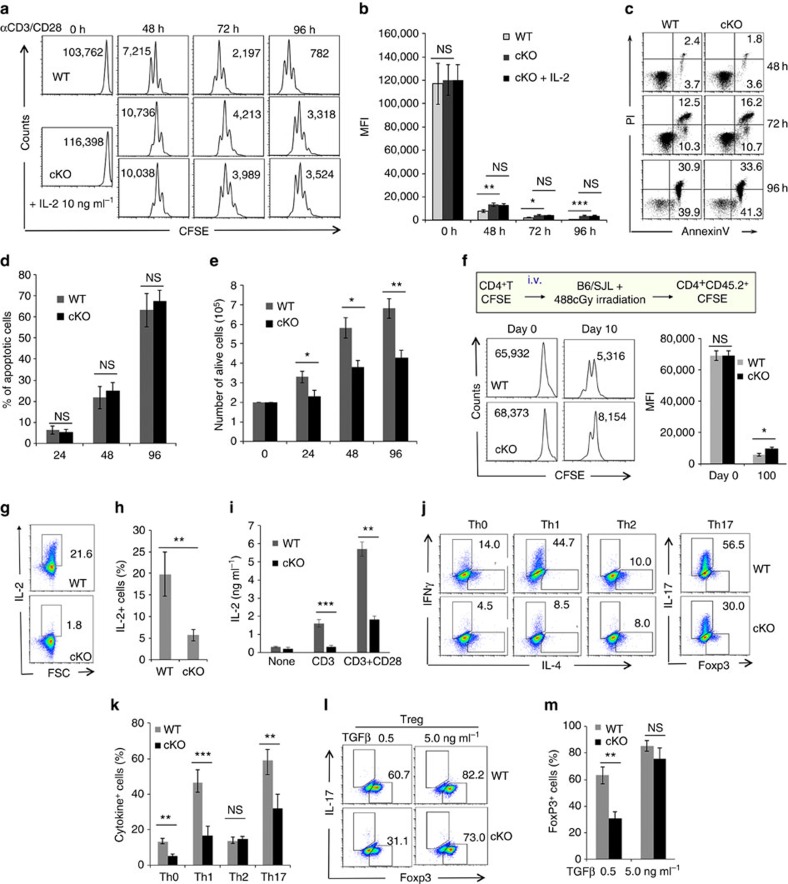
*In vitro* activation and differentiation of Hrd1 cKO T cells. (**a**–**e**) Naive CD4 T cells were purified from the spleens of WT and Hrd1 cKO mice, and stained with CFSE. Cells were then cultivated in the presence of anti-CD3 plus anti-CD28 antibodies (1 mg ml^−1^ each). Cell proliferation (**a**,**b**) and apoptosis (**c**,**d**) at each indicated time point were analysed by flow cytometry. The representative data (**a**,**c**) and the averages (**b**,**d**) from five independent experiments are shown. (**e**) The absolute numbers of dividing cells from five independent experiments are indicated. (**f**) CFSE-stained cells were adoptively transferred into B6/SJL congenic mice bearing a CD45.1 marker. Ten days after the transfer, receipt mice were killed and the CD45.2^+^ CD4 T cells were gated for their CFSE dilution. Representative images (left panels) and the averages (right panels) from three pairs of mice were shown. (**g**–**m**) Naive CD4 T cells from WT and Hrd1 cKO mice were cultivated with anti-CD3 plus anti-CD28 (**g**–**i**) or at each polarization condition (**j**–**m**). IL-2 production and Th-cell differentiation were analysed by intracellular staining as described in the materials and methods (**g**,**h**). IL-2 in the culture supernatants 3 days after anti-CD3 plus anti-CD28 stimulation was analysed by enzyme-linked immunosorbent assay (ELISA). (**i**) The representative images (**g**,**j**,**l**) and the mean±s.d. (**h**,**i**,**k**,**m**) from five independent experiments are shown. The Mann–Whitney test was used for the statistical analysis. NS, no significant difference; *P<0.05, **P<0.01 and ***P<0.001.

To study the role of Hrd1 in regulating the T-cell immune response, first we analysed Hrd1 expression in mouse CD4^+^ T cells. Hrd1 messenger RNA (mRNA) expression was relatively low in naive CD4 T cells compared with B cells (Supplementary Fig. 1a). Stimulation with anti-CD3/CD28 significantly (*P*<0.01) enhanced both Hrd1 mRNA and protein expression in CD4 T cells (Supplementary Fig. 1b,c), suggesting that Hrd1 expression is regulated in T cells by TCR/CD28 signalling. We then generated a strain of T-cell-specific Hrd1 knockout (Hrd1 cKO) mice by breeding mice with *Hrd1* alleles (Hrd1^fl/fl^)^20^ with CD4-Cre transgenic mice (Supplementary Fig. 1d). Immunoblot analysis confirmed the complete elimination of Hrd1 protein expression in purified CD4^+^ T cells from the resulting Hrd1^fl/fl^CD4-Cre mice (Hrd1 cKO mice; Supplementary Fig. 1e). By analysing cell surface CD4 and CD8 expression in the thymocytes of the Hrd1 cKO mice, we observed a slight, but statistically significant reduction in both CD4^+^CD8^−^ and CD4^−^CD8^+^ thymocytes compared with Hrd1^+/+^CD4-Cre (wild type, WT) control mice (Supplementary Fig. 2a–c), implying that Hrd1 function is involved in both CD4-positive and CD8-positive T-cell development. Consistent with this notion, we also saw a marked reduction in the frequencies, as well as the absolute numbers, of either total CD3^+^ or CD4^+^ and CD8^+^ T cells in the spleens of Hrd1 cKO mice compared with WT mice (Supplementary Fig. 2g–h). Despite an increase in the frequency of γδ T cells in Hrd1 cKO mice, their absolute numbers were not altered (Fig. 1i). Therefore, the increased frequency of γδ T cells is likely a consequence of the reduction of αβ T cells in the Hrd1 cKO mice. In addition, the percentage of Tregs (CD25^+^FoxP3^+^) was not different, but the absolute cell numbers of Tregs decreased significantly, in cKO mice compared with WT controls (Supplementary Fig. 2f,j), which appears to be due to a lower total number of T cells in the spleens of cKO mice. Hrd1 conditional deletion in T cells therefore resulted in perturbed development of thymocytes and led to lymphocytopenia in both the thymus and spleen.

### Hrd1 is required for T-cell activation and differentiation

As TCR/CD28 signalling induced Hrd1 protein and mRNA expression in T cells (Supplementary Fig. 1b,c), we hypothesized that upregulation of Hrd1 in activated T cells might play an important role in T-cell function. Indeed, Hrd1 cKO T cells showed markedly lower proliferation on *in vitro* stimulation with anti-CD3/anti-CD28 antibodies compared with WT T cells because the carboxyfluorescein succinimidyl ester (CFSE) dilution was slowed down in Hrd1-null T cells (Fig. 1a,b), and the numbers of proliferative cells were reduced (Supplementary Fig. 3a). Because Hrd1 has been identified as an anti-apoptotic factor that prevents cells from ER stress-induced apoptosis, we further determined whether Hrd1 deletion affects CD4 T-cell survival during *in vitro* activation. The percentages of annexin V-positive cells were indistinguishable between WT and Hrd1 cKO T cells (Fig. 1c,d), suggesting that the observed reduction in T-cell growth was not due to the increased apoptosis. By contrast, the cell numbers of proliferative Hrd1-null T cells were significantly reduced, confirming that Hrd1 deficiency impairs T-cell growth upon *in vitro* TCR/CD28 stimulation (Fig. 1e). The *in vivo* analysis by adoptive transfer of CFSE-stained Hrd1 cKO CD4^+^ T cells into congenic recipient mice confirmed the reduction in Hrd1 cKO T-cell homeostatic proliferation compared with WT T cells (Fig. 1f; Supplementary Fig. 3b). A similar proliferative defect was further confirmed in Hrd1-null CD8 T cells as the CFSE fluorescence intensity of Hrd1-null T cells were less diluted (Supplementary Fig. 3c,d). Next, we assessed the production of IL-2 in Hrd1 cKO CD4 T cells and WT littermate controls after stimulation with anti-CD3 or anti-CD3 plus anti-CD28 antibodies. We observed that Hrd1 cKO T cells failed to secrete IL-2 as measured by both intracellular staining (Fig. 1g,h) and enzyme-linked immunosorbent assay (Fig. 1i), indicating that Hrd1 function is required for both T-cell clonal expansion and IL-2 production. As IL-2 is a known T-cell growth factor, one possible explanation for the reduced proliferation of Hrd1 cKO T cells is impaired IL-2 production; however, addition of exogenous IL-2 failed to rescue Hrd1 cKO CD4 T-cell proliferation (Fig. 1a, bottom panel, and Fig. 1b). Therefore, reduced IL-2 production appears not to be responsible for the impaired proliferation of Hrd1 cKO T cells.

*Hrd1* gene deletion impairs T-cell activation is unlikely due to the altered expression levels of cell surface receptors, because the expression of TCR complex including TCRβ and CD3ɛ and the co-stimulatory receptor CD28 are indistinguishable between WT and Hrd1-null T cells from the spleen and draining lymph nodes (Supplementary Fig. 4a–c). Similarly, the cell surface expression levels of the early activation markers CD25 and CD69 were not altered by *Hrd1* gene deletion in naive CD4 T cells and even after 24-h stimulation (Supplementary Fig. 5a–d). Antigen recognition by TCR in the presence of co-stimulation through CD28 triggers the phosphorylation of upstream signalling molecules during early phase of T-cell activation. Loss of Hrd1 functions appears not to impair the overall tyrosine phosphorylation patterns in CD4 T cells on either TCR stimulation alone or TCR plus CD28 stimuli (Supplementary Fig. 6a). Moreover, the activation of the mitogen-activated protein kinases, including Erk1/2 and JNK1, was unaltered by Hrd1 deficiency during T-cell activation (Supplementary Fig. 6b). Therefore, Hrd1 is unlikely to promote T-cell activation through activating the upstream TCR/CD28 signalling pathway.

After clonal expansion, and depending on the cytokine environment, CD4^+^ T cells differentiate into a variety of effector subsets, including Th1, Th2, Th17 and induced Tregs^5^. To determine whether Hrd1 affects CD4 T-cell differentiation *in vitro*, we cultured CD4^+^CD25^−^ naive T cells from Hrd1 cKO and WT mice under different cytokine environments to direct them into different Th subsets, as reported previously^21^. Although Th2 differentiation was normal (Fig. 1j,k), Hrd1 cKO Th0 and Th1 cells exhibited lower production of IFN-γ compared with cells differentiated from WT controls. Meanwhile, Hrd1 cKO Th17 cells produced less IL-17 (Fig. 1j,k). While the FoxP3-positive Treg percentage at lower transforming growth factor-β (TGF-β) concentration was significantly reduced, Hrd1 deficiency did not affect Treg differentiation at a higher TGF-β concentration (Fig. 1l,m). Taken together, these data indicate that Hrd1 is a positive regulator of T-cell proliferation, activation and Th-cell differentiation *in vitro*. To further determine whether the defect in Th1 and Th17 cytokine production by Hrd1-null CD4 T cells is due to the impaired proliferation, we analysed the cell cycle-based production of IFN-γ and IL-17 by Hrd1 cKO CD4 T cells. As shown in Fig. 7, the overall IL-2, IFN-γ and IL-17 production by Hrd1-null CD4 T cells, as well as their cell cycle division, were reduced, confirming our initial observation that Hrd1 functions are required for the optimal T-cell proliferation and cytokine production. Importantly, Hrd1 cKO T cells produced a markedly less IFN-γ and IL-17 comparing with those of WT CD4 T cells even at the same cycle of cell division (Supplementary Fig. 7), indicating that the reduced cytokine production by Hrd1 cKO T cells is uncoupled with the cell cycle progression.

### Hrd1 deficiency impairs antigen-specific T-cell immunity

**Figure 2:**
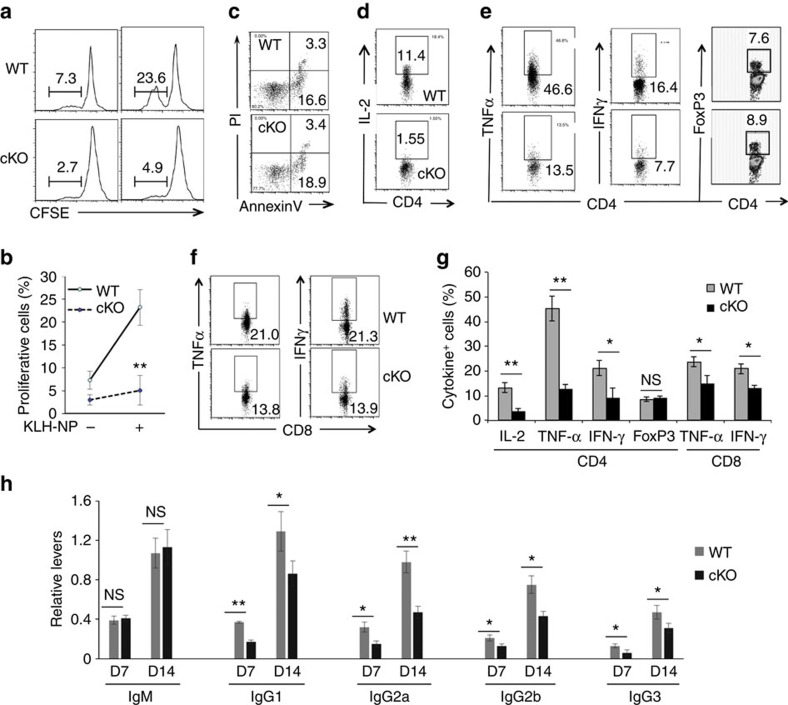
Impaired antigen-specific T-cell immune response in Hrd1 cKO mice. WT and Hrd1 cKO mice were immunized with 50 μg KLH-NP in CFA on day 1 followed by a boosted immunization with 50 μg KLH-NP in IFA on day 8. Mice were killed on day 14. (**a**–**c**) Total splenocytes were stained with CFSE and cultivated with 100 ng ml^−1^ of KLH-NP for 5 days. The proliferation (**a**,**b**) and apoptosis (**c**) of CD4^+^ T cells were analysed. The representative images (**a**,**c**) and data from five pairs of mice (**b**) are shown. (**d**–**g**) The production of IL-2 in CD4 T cells (**d**), and TNF-α, IFN-γ and FoxP3^+^ Tregs in CD4 (**e**) and CD8 (**f**) cells were analysed. The representative data (**d**–**f**) and the mean±s.d. (**g**) from five pairs of mice are shown. (**h**) The levels of NP-specific antibodies in the sera of immunized Mann–Whitney test mice were analysed by enzyme-linked immunosorbent assay (ELISA). Error bars represent data from five pairs of mice (mean±s.d.). The Mann–Whitney test was used for the statistical analysis. NS, no significant difference; *P<0.05, **P<0.01 and ***P<0.001.

To determine the effect of Hrd1 deficiency on T-cell activation *in vivo*, we immunized 8–10-week-old Hrd1 cKO mice and their WT littermate controls with 4-hydroxy-3-nitrophenyl acetyl-keyhole limpet haemocyanin (NP-KLH) in complete Freund's adjuvant (CFA) on day 1 and a boosted immunization of NP-KLP in incomplete Freund's adjuvant (IFA) on day 8. Immunized mice were killed on day 14 and the NP-specific T-cell immune response was analysed. Consistent with our *in vitro* observations, there was a significant decrease in the proliferation of Hrd1 cKO T cells on cultivation with NP antigen (Fig. 2a,b). This reduction in NP-specific T-cell growth was not due to an increase in activation-induced cell death, because WT and Hrd1 cKO CD4 T cells derived from splenocytes showed similar amounts of apoptosis (Fig. 2c). Furthermore, IL-2, TNF-α and IFN-γ produced by CD4^+^ T cells and TNF-α and IFN-γ produced by CD8^+^ T cells were markedly decreased in Hrd1 cKO mice compared with controls (Fig. 2d–g), suggesting that Hrd1 functions as a regulator required for antigen-specific T-cell activation *in vivo*. In addition, similar to those observed in mice under naive conditions, the percentage of CD25^+^FoxP3^+^ Tregs was not altered in the spleens of immunized mice (Fig. 2e,g)

To determine the effects of Hrd1 on the T-cell-dependent humoral immune response, we measured NP-specific antibody levels in sera from mice 7 days after the first immunization with NP-KLH/CFA (the primary immune response) and again after the boosted immunization with NP-KLP/IFA. As shown in Fig. 2h, Hrd1 cKO mice exhibited lower antigen-specific antibodies of IgG1, IgG2a, IgG2b and IgG3 in the primary and secondary responses compared with WT controls. In contrast, the levels of NP-specific IgM were not altered. These results indicate that Hrd1 is required for the T-cell-dependent humoral response.

### Hrd1 interacts with p27^kip1^ in T cells

**Figure 3:**
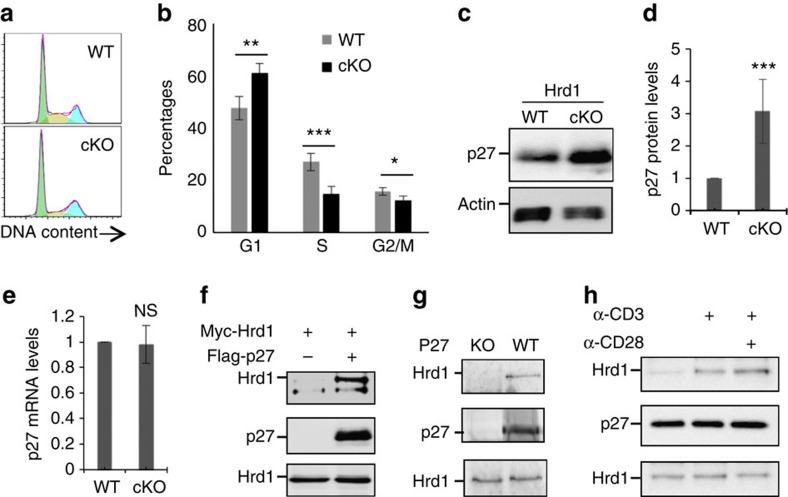
Hrd1 interacts with p27^kip1^ in T cells. WT and Hrd1 cKO CD4 T cells were stimulated with anti-CD3 plus anti-CD28 (2 μg ml^−1^) for 24 h. (**a**,**b**) Cell cycle was analysed by DNA staining and flow cytometry. Representative images (**a**) and data from five independent experiments (**b**) are shown. (**c**–**e**) p27^kip1^ protein (**c**,**d**) and mRNA (**e**) levels were analysed by immunoblotting and real-time PCR with reverse transcription. Representative images (**c**) and data from five independent experiments (**d**) are shown. (**f**) HEK293 cells were transfected with Myc-tagged Hrd1 and Flag-p27. The interaction of Hrd1 with p27^kip1^ in the transiently transfected cells was determined by co-immunoprecipitation with anti-Flag and the bound Hrd1 was detected by immunoblotting with anti-Myc antibody (top panel). The same membrane was re-blotted with anti-Flag antibody (middle panel) and the expression levels of Hrd1 in the whole-cell lysates were confirmed by immunoblotting (bottom panel). (**g**) CD4 T cells from WT and p27^kip1^ KO mice were stimulated with anti-CD3 plus anti-CD28 for 24 h. The interaction of p27^kip1^ with Hrd1 was analysed by immunoprecipitation with anti-p27 and immunoblotting with anti-Hrd1 antibody (top panel). The expression levels of p27 (middle panel) and Hrd1 (bottom panel) in whole-cell lysate was confirmed by immunoblotting. (**h**) CD4 T cells were stimulated with anti-CD3 or anti-CD3 plus anti-CD28 for 30 min, and Hrd1 interaction with p27^kip1^ was determined as in **g**. Error bars (**b**,**d**,**e**) represent data from five pairs of mice (mean+s.d.). Mann–Whitney test was used for the statistical analysis. NS, no significant difference; *P<0.05, **P<0.01 and ***P<0.001.

Our finding that Hrd1 deficiency impaired T-cell proliferation without increasing activation-induced cell death *in vitro* and in mice implies an important role for Hrd1 in regulating T-cell cycle entry during clonal expansion. To test this hypothesis, we analysed the cell cycle progression of Hrd1 cKO and WT T cells on TCR/CD28 stimulation. As indicated in Fig. 3a,b, Hrd1 deficiency resulted in a partial cell cycle arrest at the G1–S transition because of a significant increase in G1/G0 phase cells and a reduction in cells at S and G2/M phases. p27^kip1^ has been shown to negatively regulate the transition from G1 to S phase by inhibiting the activity of CDK2 (refs 22, 23, 24). As a really interesting new gene (RING) finger-containing E3 ubiquitin ligase, Hrd1 often downregulates the expression levels of proteins through ubiquitination-mediated degradation^18^,^19^,^20^. We speculated that Hrd1 may regulate CD4 T-cell proliferation by ubiquitinating p27^kip1^ for subsequent destruction. We found that p27^kip1^ protein expression (Fig. 3c,d), but not mRNA level (Fig. 3e), was significantly increased in Hrd1 cKO T cells, implying that Hrd1 promotes cell cycle progression through p27^kip1^ protein destruction. To determine whether Hrd1 is an E3 ligase of p27^kip1^, we first asked whether Hrd1 physically interacts with p27^kip1^. Co-immunoprecipitation and immunoblot analysis detected a physical interaction between Hrd1 and p27^kip1^ proteins; Hrd1 protein was detected in the immunoprecipitates from HEK293 cells transiently transfected with both Hrd1 and p27^kip1^ expression plasmids, but not from those transfected with Hrd1 alone (Fig. 3f). The interaction between endogenous Hrd1 and p27^kip1^ in mouse primary CD4 T cells was confirmed by detection of the Hrd1 protein in the anti-p27^kip1^ immunoprecipitates from WT but not p27^kip1^-null T cells, clearly demonstrating that p27^kip1^ is an interacting protein of Hrd1 in T cells (Fig. 3g). The interaction of Hrd1 with p27^kip1^ occurred at low levels in naive T cells, and stimulation with anti-CD3 for 30 min resulted in a significant increase in their interaction, which was further enhanced by addition of anti-CD28 (Fig. 3h). These results indicate that Hrd1/p27^kip1^ interaction is regulated by TCR/CD28 signalling.

### Hrd1 is an E3 ubiquitin ligase of p27^kip1^ in T cells

**Figure 4:**
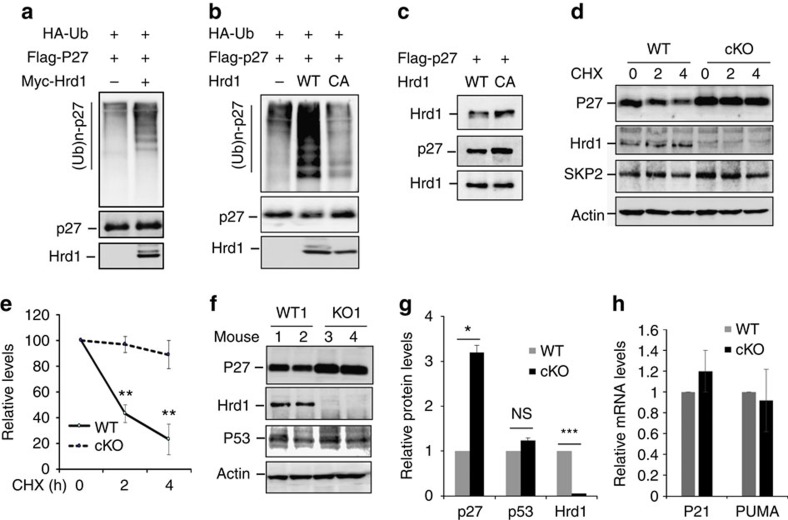
Hrd1 is an E3 ubiquitin ligase of p27^kip1^. (**a**,**b**) Flag-p27 and HA-Ub expression plasmids were co-transfected with Myc-Hrd1 or its CA mutant. p27^kip1^ protein ubiquitination was determined by immunoprecipitation of p27^kip1^ protein and immunoblotting with anti-HA antibodies (top panels). The same membranes were re-probed with anti-Flag (middle panels) and Hrd1 expression in the whole-cell lysates was confirmed by immunoblotting with anti-Myc antibody (bottom panels). (**c**) The interaction of p27 with both WT and Hrd1/CA mutant in transiently transfected 293 cells were analysed. (**d**,**e**) WT and Hrd1 cKO CD4 T cells were cultured with anti-CD3 plus anti-CD28 overnight and then chased by cycloheximide (CHX) for the indicated times. The protein expression levels of p27, Hrd1, SKP2 and the loading control β-actin were examined by immunoblotting using each specific antibody. Representative images (**d**) and quantifications of five independent experiments (**e**) are shown. (**f**–**h**) The protein expression levels of p27^kip1^, Hrd1, p53 and β-actin in WT and Hrd1 cKO CD4 T cells were determined by immunoblotting (**f**), and the average levels from five pairs of mice are indicated (**g**). Total RNA was extracted from stimulated cells and the expression levels of p21 and PUMA were analysed by real-time PCR with reverse transcription (**h**). Mann–Whitney test was used for the statistical analysis. NS, no significant difference; *P<0.05, **P<0.01 and ***P<0.001.

We next examined whether Hrd1 catalyses ubiquitination of p27^kip1^. Anti-HA antibody detected bands with gradually increasing molecular weights in anti-p27^kip1^ immunoprecipitates from cells transiently transfected with p27^kip1^ and HA-ubiquitin expression plasmids, indicating that p27^kip1^ is ubiquitinated. Hrd1 co-expression led to a substantial increase in p27^kip1^ ubiquitination (Fig. 4a). The E3 ligase activity of Hrd1 is required for p27^kip1^ ubiquitination, because co-expression of Hrd1 containing an alanine point mutation at the critical cysteine within the RING E3 ligase region (Hrd1/CA)^25^ failed to enhance p27^kip1^ ubiquitination (Fig. 4b). Interaction between p27^kip1^ with Hrd1 and the Hrd1/CA mutant was comparable (Fig. 4c). Together, these results indicate that Hrd1 is an E3 ubiquitin ligase that targets p27^kip1^.

Our observation that p27^kip1^ protein but not its mRNA levels were elevated in Hrd1 cKO T cells suggests that Hrd1-mediated p27^kip1^ ubiquitination is likely to promote p27^kip1^ protein destruction. To test this notion, we measured the half-life of p27^kip1^ in CD4 T cells chased with the translational inhibitor cycloheximide (CHX). p27^kip1^ was gradually degraded in Hrd1^+/+^ CD4 T cells following CHX treatment (Fig. 4d), with a half-life of ∼1.5 h (Fig. 4e). In contrast, the protein expression levels of p27^kip1^ were significantly higher in Hrd1 cKO CD4 T cells following CHX, with a half-life extended to beyond 4 h (Fig. 4e), indicating that Hrd1 E3 ligase is responsible for p27^kip1^ protein degradation in T cells. The S-phase kinase-associated protein 2 (SKP2), an F-box protein, has been well characterized as an E3 ubiquitin ligase targeting p27^kip1^ for ubiquitination and degradation^26^,^27^,^28^. It is possible that Hrd1 controls p27^kip1^ protein stability through SKP2 in T cells, with decreased expression of SKP2 in Hrd1 cKO CD4 T cells leading to p27^kip1^ accumulation. However, SKP2 protein levels in WT and Hrd1 cKO T cells were indistinguishable (Fig. 4d), excluding the possibility that p27^kip1^ protein accumulation in Hrd1-null T cells is due to the reduced SKP2 expression. In addition, p53 has been identified as a substrate of Hrd1, and it is possible that loss of Hrd1 results in T-cell hypo-proliferation through p53 protein accumulation^18^. To test this possibility, we examined the expression level of p53 protein in WT and Hrd1 cKO mice. Neither the expression level of p53 protein nor the transcription levels of its target genes, p21 and PUMA^29^,^30^, were altered by *Hrd1* gene deletion in T cells (Fig. 4f–h). Collectively, our study identifies Hrd1 as a novel E3 ubiquitin ligase of p27^kip1^ in T cells.

### Hrd1 promotes T-cell activation through p27^kip1^

**Figure 5:**
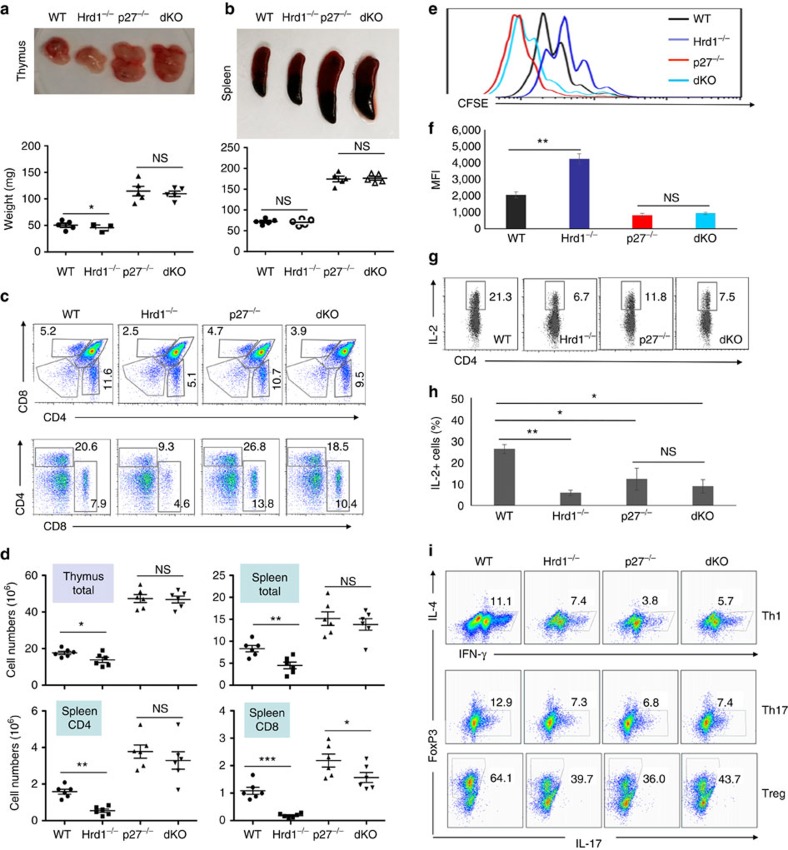
Hrd1 regulates T-cell activation partially through p27^kip1^. (**a**,**b**) Thymus (**a**) and spleen (**b**) were isolated from 8–10-week-old WT, Hrd1 cKO, p27^kip1^ KO and double KO (dKO) mice. Representative images (top panels) and the weights in milligrams (mg; bottom panels) are shown. (**c**,**d**) Single-cell suspensions from the thymus and spleen were analysed by cell surface staining of CD4 and CD8. Representative flow data (**c**) and the absolute numbers of each indicated population from five sets of mice (**d**) are shown. (**e**–**h**) Naive CD4 T cells from WT, Hrd1 cKO, p27^kip1^ KO and dKO mice were stained with CFSE and cultured with anti-CD3 plus anti-CD28. Cell proliferation (**e**,**f**) and IL-2 production (**g**,**h**) were determined. Representative images (**e**,**g**) and data from five independent experiments are shown (**f**,**h**). (**i**) Naive CD4 T cells from WT, Hrd1 cKO, p27^kip1^ KO and dKO mice under Th1, Th17 or Treg polarization conditions. The production of each indicated cytokines or FoxP3 expression was analysed by intracellular staining. Representative images are shown here and the results from five independent experiments are indicated in Supplementary Fig. 8. Mann–Whitney test was used for the statistical analysis. NS, no significant difference; *P<0.05, **P<0.01 and ***P<0.001.

p27^kip1^ plays a critical role in regulating T-cell clonal expansion, differentiation and memory development, and its upregulation is known as an important molecular mechanism in T-cell peripheral tolerance induction^4^,^22^,^31^,^32^. On the basis of our discoveries that Hrd1 is an E3 ubiquitin ligase of p27^kip1^ and loss of Hrd1 leads to p27^kip1^ protein accumulation in T cells, we speculated that Hrd1 may regulate T-cell immune response through p27^kip1^ degradation. We predicted that further deletion of p27^kip1^ in Hrd1 cKO T cells would rescue T-cell activation and differentiation from the Hrd1 deficiency defect. Therefore, we crossed Hrd1^−/−^CD4-cre mice with p27^kip1−/−^ KO mice to create Hrd1^−/−^p27^kip1−/−^CD4-cre double-knockout (DKO) mice. Consistent with the previous observations, both the thymus and spleen sizes were enlarged in p27^kip1^ KO mice. Notably, in contrast to a slight reduction in the thymus and spleen sizes in Hrd1 cKO mice, the DKO animals exhibited a phenotype identical to that of p27^kip1^ KO mice, with the enlarged thymus and spleens (Fig. 5a,b), and increased total cell numbers, frequencies and absolute cell numbers of CD4 T cells in both the spleen and thymus (Fig. 5c,b). Moreover, while DKO mice showed a modest but statistically significant decrease in CD8 absolute cell numbers in the spleen compared with p27^kip1^ KO mice (Fig. 5d), indicating that p27^kip1^ deletion resulted in a partial rescue of the reduction in CD8 T cells. Importantly, DKO T cells exhibited the same proliferation rate as p27^kip1^ KO T cells on TCR/CD28 stimulation (Fig. 5e,f) as the numbers of proliferative p27^kip1^ KO and p27^kip1^Hrd1 DKO cells are comparable (Supplementary Fig. 8a), clearly indicating that Hrd1 promotes T-cell growth in a p27^kip1^-dependent manner. Collectively, our observations indicate that Hrd1 regulates T-cell development and proliferation through catalysing p27^kip1^ protein degradation.

Intracellular staining analysis detected comparable levels of IL-2 production in Hrd1 cKO and Hrd1/p27^kip1^ DKO T cells on *in vitro* TCR/CD28 stimulation (Fig. 5g,h), indicating that Hrd1 promotes IL-2 expression in a p27^kip1^-independent manner. Similarly, the levels of both IFN-γ and IL-17 production under Th1 and Th17 polarization conditions, as well as the percentages of FoxP3-positive Tregs, were indistinguishable between Hrd1 cKO and Hrd1/p27^kip1^ DKO T cells (Fig. 5i; Supplementary Fig. 8b). Consistent with previous studies^31^,^32^, we confirmed a marked reduction in IL-2 production and Th1, Th17 and Treg differentiation of p27^kip1^ KO CD4 T cells (Fig. 5g–i; Supplementary Fig. 8). Together, these data indicate that Hrd1-mediated p27^kip1^ protein degradation, while necessary for T-cell development and differentiation, is dispensable for IL-2 production and differentiation into Th1, Th17 and Tregs.

### T-cell-specific Hrd1 deletion protects mice from EAE

**Figure 6:**
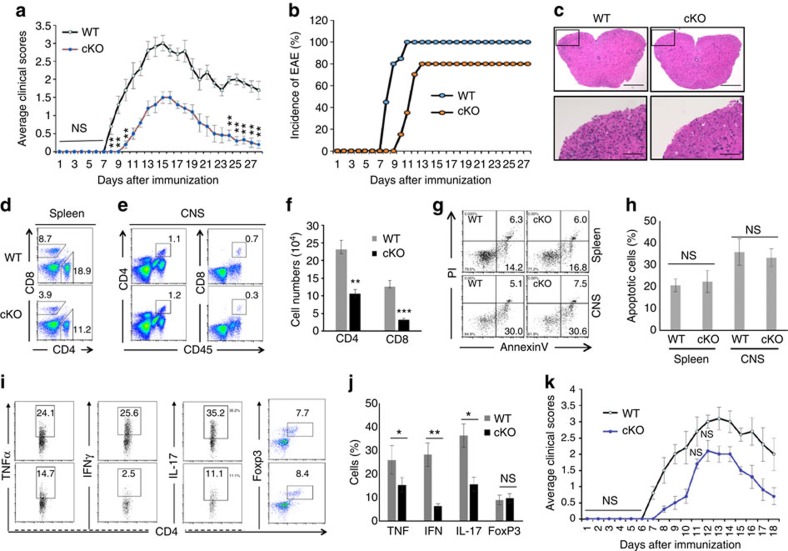
EAE induction in Hrd1 cKO mice. (**a**,**b**) EAE was induced in 8–10-week-old WT and Hrd1 cKO mice, and the symptoms were scored daily. The average symptom scores (**a**) and disease incidence (**b**) from 15 pairs of mice are shown. (**c**–**j**) The spinal cords were isolated 12–15 days after EAE induction. Representative images of H&E staining are shown; scale bars, 200 μm (top); 10 μm (bottom; **c**). The splenocytes (**d**) and infiltrated lymphocytes were isolated and analysed by flow cytometry for each of the indicated cell surface markers (**d**,**e**) and apoptosis markers (**g**). The cytokine production was determined by intracellular staining (**h**,**j**). Error bars represent data from five independent isolation (each purification was from spinal cords pooled from three mice; **f**,**h**,**j**). (**i**) Purified naive CD4 T cells from WT and Hrd1 cKO mice were adoptively transferred into T-cell-null mice. Two days after transfer, EAE induction was performed and the mice were scored for disease development. Error bars represent data from five pairs of recipient mice. Mann–Whitney test was used for the statistical analysis. *P<0.05, **P<0.01 and ***P<0.001. The *P* value is <0.05 in (**a**,**k**) at each scored time point unless specifically indicated.

Our findings that Hrd1 is a positive regulator of the T-cell activation and differentiation prompted us to test its therapeutic potential in autoimmune disease. We induced an experimental form of autoimmune encephalomyelitis (EAE), a model of human multiple sclerosis, in WT and Hrd1 cKO mice, and assessed the effect of genetic Hrd1 suppression in T cells, as reported previously^33^. When immunized with myelin oligodendrocyte glycoprotein peptide (MOG_35–55_), WT C57/B6 mice developed clinical symptoms of EAE starting on day 7 after immunization that peaked around day 14, followed by a partial recovery phase. In contrast, disease onset in Hrd1 cKO mice was delayed for at least 2 days, with a statistically dramatic reduction in symptoms (Fig. 6a). In addition, 100% of WT mice developed EAE disease by day 10 after MOG immunization, while only 80% of Hrd1 cKO mice developed disease and 20% of mice were fully protected from MOG-induced autoimmune disease (Fig. 6b). Histological analysis of the central nervous system (CNS) tissues isolated from MOG-immunized mice on day 14 showed fewer infiltrated lymphocytes into the spinal cord in Hrd1 cKO mice (Fig. 6c), indicating that genetic Hrd1 deletion suppresses the MOG-induced autoimmune response in mice.

We then purified the infiltrated lymphocytes in brain and spinal cord tissues isolated from MOG-immunized mice during peak EAE symptoms. As expected, Hrd1 cKO mice showed a significant decrease in the percentage and absolute number of CD4^+^ and CD8^+^ T cells in the spleen (Fig. 6d) and CNS (Fig. 6e,f). Again, the reduction in infiltrated T cells during disease development is not likely to be due to an elevation in apoptosis, because the percentages of annexin V-positive CD4^+^ T cells isolated from CNS tissues of Hrd1 cKO mice and their WT control littermates were comparable (Fig. 6g,h). The Hrd1 cKO mice exhibited a significant decrease in the proportion of TNF-α^+^, IFN-γ^+^ and IL-17^+^CD4^+^ cells, but no difference in the percentage of CD4^+^FoxP3^+^ cells compared with WT control mice (Fig. 6i,j). Consistent with the results observed in naive mice, a significant reduction in both CD4 and CD8 T cells in the spleens of Hrd1 cKO mice were detected after MOG immunization (Fig. 6d), raising the possibility that the decreased number of T cells in Hrd1 cKO mice is responsible for protecting mice from the disease. To test this, we purified CD4 T cells from WT and Hrd1 cKO mice, and adoptively transferred an equal number of them to T-cell-null mice. One day after transfer, EAE induction was performed. As shown in Fig. 6k, Hrd1 cKO T-cell recipient mice showed resistance to EAE as indicated by modest symptoms and delayed onset of disease, suggesting that Hrd1 deletion in T cells protected the mice from MOG-induced EAE. Taken together, these results support the notion that Hrd1 is a potential target for autoimmune disease treatment.

### Hrd1 potentially promotes human autoimmunity

**Figure 7:**
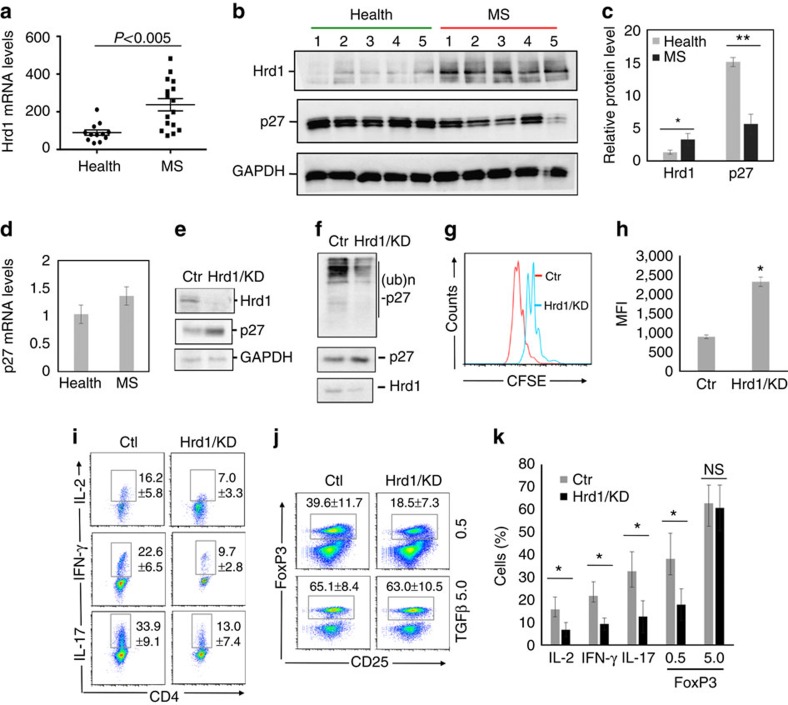
Analysis of Hrd1 expression and functions in human CD4 T cells. (**a**) The levels of Hrd1 expression in freshly isolated CD4 T cells from healthy donors (*N*=12) or MS patients (*N*=16) by real-time PCR. (**b**–**d**) CD4 T cells freshly isolated CD4 T cells from healthy donors or MS patients (*N*=5) were lysed and subjected to western blotting analysis for the protein levels of Hrd1 and p27^kip1^ using GAPDH as a loading control (**b**), and the relative band intensities were quantified (**c**). The mRNA levels of p27^kip1^ in samples of (**b**) were determined by real-time PCR using β-actin as a control (**d**). (**e**–**k**) Purified CD4 T cells from healthy donors were infected with lentivirus carrying control or Hrd1-specific shRNA. (**e**,**f**), green fluorescent protein (GFP)-positive cells were sorted, the expression levels of Hrd1, p27^kip1^ and GAPDH were examined by western blotting (**e**), and p27^kip1^ ubiquitination was determined as in Fig. 4a (**f**). (**g**,**h**) Cells were stained with far-red cell trace and cultivated for 3 days with TCR/CD28 stimulation. GFP-positive cells were gated and the proliferation was analysed for CFSE dilution. (**h**,**i**) Cells were cultivated under Th1, Th17 and Treg polarization conditions and the gated GFP^+^ cells were analysed. Representative images (**g**,**i**,**j**) and the percentages from three independent experiments (**h**,**k**) are shown. Mann–Whitney test was used for the statistical analysis in **a**,**c**,**d**,**h** and **k**.

The fact that Hrd1 expression is upregulated during T-cell activation and that Hrd1 enhances autoimmune response in mice promoted us to test whether Hrd1 expression is associated with human autoimmune diseases. Indeed, analysis the expression levels of Hrd1 detected a significant increase in Hrd1 mRNA expression levels CD4 T cells from the multiple sclerosis (MS) patients than that of healthy controls (Fig. 7a), suggesting a pathogenic role of Hrd1 elevation in human MS. We then further confirmed the marked increase in Hrd1 protein expression levels in CD4 T cells from MS patients (Fig. 7b,c). Notably, in contrast to the elevated Hrd1 protein expression levels, p27^kip1^ protein, but not its mRNA expression levels are decreased in CD4 T cells from MS patients comparing with healthy controls (Fig. 7b–d), suggesting that Hrd1 regulates p27^kip1^ protein stability in human CD4 T cells and that Hrd1 promotes CD4 T-cell autoimmunity through p27^kip1^ protein destruction. To support this conclusion, we used a short hairpin RNA (shRNA) and knocked down Hrd1 expression in human CD4 T cells, and demonstrated that Hrd1 knockdown resulted in a significant increase in p27^kip1^ protein expression (Fig. 7e). In addition, knockdown of Hrd1 expression largely inhibited p27^kip1^ ubiquitination in human CD4 T cells (Fig. 7f). These results confirm that Hrd1 is an E3 ubiquitin ligase in human T cells. We then examined the effect of Hrd1 suppression on the activation and differentiation of human CD4 T cells. As expected, Hrd1 knockdown in human CD4 T cells inhibited their proliferation, IL-2 production, Th1 and Th17 differentiation (Fig. 7g–k; Supplementary Fig. 8c). Moreover, similar to that in mouse CD4 T cells, Hrd1 suppression slightly impaired Treg polarization at low but not high concentrations of TGF-β (Fig. 7j,k). Taken together, these studies demonstrate that Hrd1 is a positive regulator of human CD4 T-cell activation and differentiation, and that the elevated Hrd1 expression is involved in human autoimmune disease.

**Figure 8:**
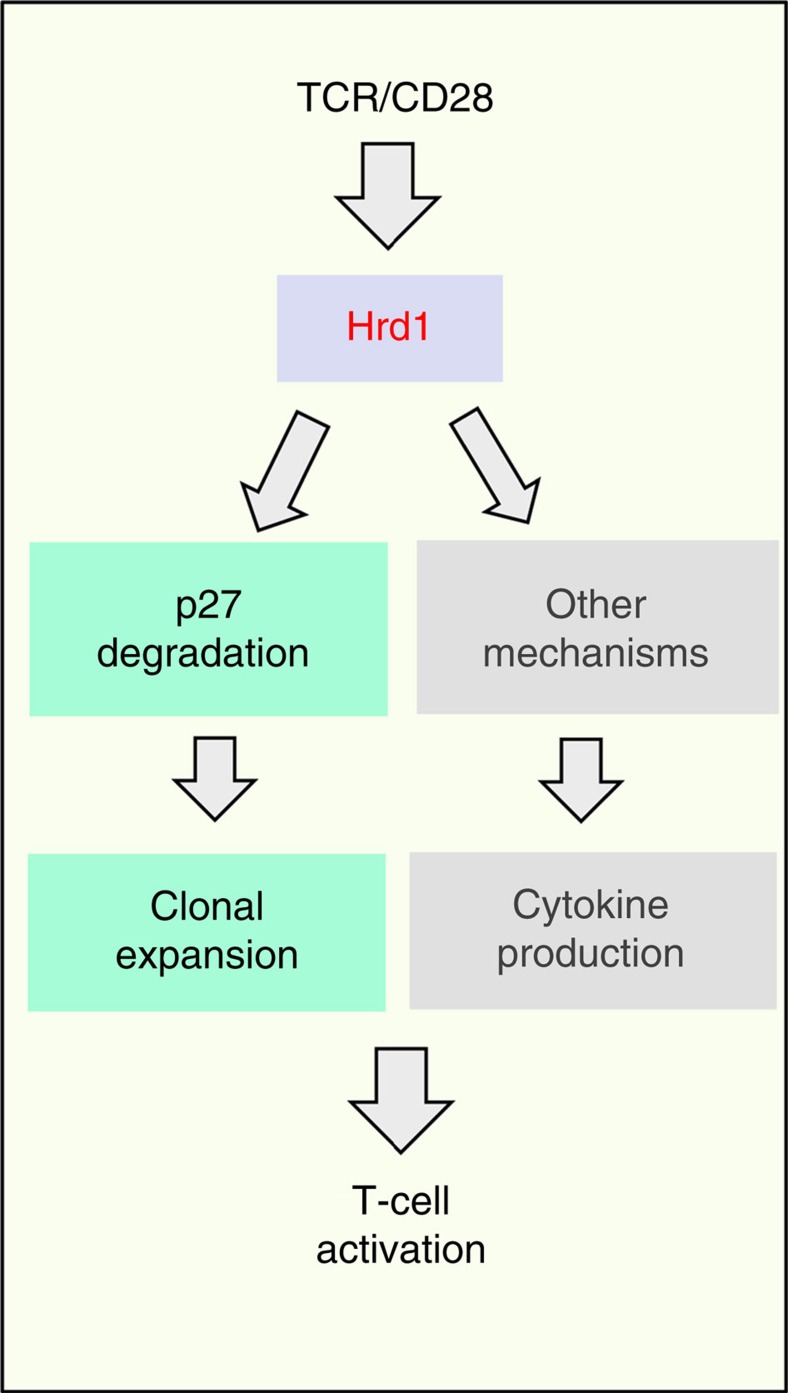
A proposed model for Hrd1 in T-cell activation. Hrd1 promotes T-cell clonal expansion through p27 degradation and regulates T-cell cytokine production through a molecular mechanism to be defined.

In summary, our study here reveals Hrd1 as a previously unappreciated positive regulator of T-cell immunity (Fig. 8). At the molecular level, Hrd1 functions as an E3 ubiquitin ligase of the CDK inhibitor p27^kip1^ and promotes the cell cycle progression through p27^kip1^ destruction during T-cell clonal expansion. However, this Hrd1-mediated p27^kip1^ degradation is unlikely responsible for Hrd1-mediated IL-2 production nor for Th1 and Th17 differentiation, because further deletion of p27^kip1^ in Hrd1-null T cells rescued T-cell growth, but not IL-2, IFN-γ and IL-17 production.

## Discussion

Our study shows that the ER membrane-spanning E3 ubiquitin ligase Hrd1 is a previously unidentified positive regulator of T cells. This conclusion is supported by the following observations: (i) targeted deletion of the *Hrd1* gene in developing thymocytes resulted in a dramatic reduction in the mature T-cell population in the thymus and spleens; (ii) Hrd1 functions were required for the proliferation and differentiation of CD4 T cells into Th1, Th17 and Tregs both *in vitro* and in mice during antigen-specific immune response; (iii) loss of Hrd1 functions led to cell cycle arrest and the accumulation of p27^kip1^ protein, an inhibitor of CDK, in T cells, and Hrd1 acted an E3 ubiquitin ligase of p27^kip1^; (iv) further genetic deletion of p27^kip1^ in Hrd1-null T cells rescued T-cell proliferation but not CD4 T-cell differentiation; (v) genetic Hrd1 suppression in T cells protected mice from MOG-induced experimental autoimmune EAE; and (vi) Hrd1 is a positive regulator of human CD4 T-cell activation and differentiation, and that the elevated Hrd1 expression are involved in human autoimmune disease multiple sclerosis.

It has been well established that downregulation of p27^kip1^ is essential for T-cell clonal expansion, the failure of which leads to T-cell anergy^31^,^32^,^34^,^35^,^36^. In the current study, we discovered that Hrd1-mediated ubiquitination and degradation of p27^kip1^ is a previously unappreciated molecular mechanism regulating cell cycle progression during the clonal expansion of T cells, as loss of Hrd1 functions led to cell cycle arrest at G1/G0 and p27^kip1^ protein accumulation. Importantly, Hrd1 interacted with p27^kip1^ in T cells and catalysed p27^kip1^ ubiquitination through its E3 ligase activity. In Hrd1-null T cells, p27^kip1^ protein accumulation does not appear to be related to the activity of SKP2, a well-characterized E3 ubiquitin ligase of p27^kip1^ (refs 26, 27, 28), because protein expression levels of SKP2 were not altered in Hrd1 cKO T cells. In addition to p27^kip1^, Hrd1 has been shown to catalyse ubiquitination and degradation of p53, a transcription factor involved in regulating gene transcription, cell cycle regulation and apoptosis^18^, raising the possibility that Hrd1 may positively regulate T-cell immunity through p53 degradation. However, the level of p53 protein and the transcription levels of its target genes, p21 and PUMA, were not affected by *Hrd1* gene deletion in T cells. Together with our findings that Hrd1 suppression did not lead to an increase in apoptosis upon *in vitro* TCR/CD28 stimulation or in mice immunized with specific antigens, p53 is unlikely to be a Hrd1 target, and p53 degradation is likely not involved in Hrd1-mediated promotion of T-cell proliferation. Therefore, our study identifies Hrd1 as an E3 ligase of p27^kip1^ and establishes that Hrd1-mediated p27^kip1^ degradation plays an important role in T-cell immunity.

In addition to its functions in positively regulating T-cell growth, Hrd1 appears to play important roles in CD4 T-cell differentiation, since the targeted deletion of Hrd1 in T cells impaired *in vitro* Th1, Th17 and Treg differentiation. In support of this notion, we confirmed a reduction in Th1 and Th17 cells in Hrd1 cKO mice immunized with specific antigens or during EAE development. Despite comparable percentages of FoxP3-positive Treg populations in WT and Hrd1 cKO mice, there was a significant reduction in the absolute T-cell numbers in Hrd1 cKO mice, both under the naive condition and during inflammation. However, Hrd1-mediated p27^Kip1^ ubiquitination is unlikely to be the only molecular mechanism underlying Hrd1 function in CD4 differentiation because further deletion of p27^kip1^ in Hrd1 cKO T cells failed to rescue the production of IL-2 and differentiation of Th1, Th17 and Treg. We recently discovered that Hrd1 is an E3 ubiquitin ligase of Blimp1, a transcriptional suppressor known to inhibit IL-2 production and Th1/Th17 differentiation^37^,^38^,^39^, raising the possibility that Hrd1 enhances IL-2 production and the differentiation of Th1 and Th17 by CD4 T cells^20^. Therefore, Hrd1-mediated Blimp1 ubiquitination and degradation to increase IL-2 production could be another possible molecular mechanism by which Hrd1 modulates CD4 T-cell differentiation. It is well established that one E3 ubiquitin ligase often targets multiple substrates to achieve its biological functions^40^, it will not be a surprise that additional substrates of Hrd1 exist in T cells to regulate the different aspects of T-cell functions such as cytokine production. Future studies are needed to illuminate whether Hrd1 functions as a positive regulator of T-cell differentiation through Blimp1 and if not, to identify the additional substrates/pathways that are regulated by Hrd1.

Hrd1 has been identified as an anti-apoptotic factor that protects cells from ER stress-induced apoptosis^41^,^42^, raising the possibility that loss of Hrd1 might cause T-cell death. In our study, the percentages of annexin V-positive apoptotic cells were indistinguishable between WT and Hrd1 cKO T cells on *in vitro* TCR/CD28 stimulation or in mice during autoimmune disease development. We recently reported that Hrd1 can promote degradation of IRE1α^14^, suggesting a possible role of Hrd1-mediated IRE1 in regulating T-cell activation and differentiation. Indeed, we detected a significant increase in the expression levels of IRE1 protein but not mRNA in Hrd1 cKO T cells, further confirming our previous finding that Hrd1 is an E3 ubiquitin ligase of IRE1. However, neither genetic nor pharmacological IRE1α inhibition had an effect on CD4 T-cell proliferation and differentiation into Th1, Th17 and Tregs^21^, excluding the possibility that Hrd1-mediated ubiquitination contributes to these processes. In addition to Hrd1, ER stress sensors and chaperons have been shown to be involved in regulation a variety of immune functions^43^,^44^,^45^.

Our study indicates that Hrd1 is a potential therapeutic target for autoimmune disease treatment because genetic suppression of Hrd1 in T cells protected mice from MOG-induced EAE. The reduction in T-cell numbers in the peripheral lymphoid organs of Hrd1 cKO mice might have contributed to disease protection; however, the impaired T-cell activation and differentiation into Th1 and Th17, without reducing the percentage of Tregs, is likely to be responsible for the protective effect in Hrd1 cKO mice. Recipient mice that received an equal number of transfer CD4 T cells showed fewer symptoms of EAE after MOG immunization. In addition, we previously showed that Hrd1 programs dendritic cells to prime CD4 T cells by facilitating MHC II transcription, and that suppression of dendritic cell Hrd1 functions is also protective against EAE development^20^. Importantly, our data show that Hrd1 upregulation appears to be associated with human autoimmunity and that suppression of Hrd1 expression in human T cells inhibited their activation and differentiation. Since TCR/CD28 stimulation induces Hrd1 expression, the possibility that this elevated Hrd1 expression in T cells is a consequence of an increase in auto-reactive T-cell activation during MS cannot be fully excluded and further studies are needed. Nevertheless, we demonstrated that this elevated Hrd1 expression is associated with the reduced p27^Kip1^ protein but not mRNA levels. Together with our observations that Hrd1 knockdown resulted in the elevated p27^Kip1^ expression and reduced p27^Kip1^ ubiquitination, our studies demonstrate that Hrd1-mediated p27^Kip1^ protein degradation as a novel molecular mechanism in human T-cell proliferation. In addition, Hrd1 suppression by shRNA-mediated knockdown inhibited human T-cell proliferation, IL-2 production, and Th1 and Th17 differentiation. Therefore, targeting Hrd1 may be beneficial for the treatment of autoimmune diseases, in particular those mediated by Th1 and Th17 cells, such as multiple sclerosis and RA. Notably, a specific small-molecule inhibitor, LS-102, has been identified recently that suppresses the E3 ubiquitin ligase catalytic activity of Hrd1 (ref. 46). Administration of LS-102 significantly reduced the symptoms of arthritis in mice immunized with collagen by inducing the apoptosis of synovial fibroblasts during synovitis^46^. It will be interesting to further investigate whether LS-102, in addition to its effects on inducing synovial fibroblast apoptosis, suppresses T-cell activation and CD4 T-cell differentiation into Th1 and Th17 in mice during collagen-induced arthritis as well as MOG-induced EAE.

## Methods

### Mice

The *Hrd1* floxed mice were used as reported^20^ and the T-cell-specific *Hrd1*-null mice (Hrd1 cKO) were generated by breeding *Hrd1* floxed mice with *CD4-Cre* transgenic mice (Jackson laboratory, Bar Harbor, ME, catalogue no. 017336). Both p27^kip1^ KO (catalogue no. 002781) and T-cell-null mice (catalogue no. 002118) were purchased from Jackson Laboratory. All the mice used in this study are in the C57/B6 genetic background and were maintained/used at the Northwestern University mouse facility under pathogen-free conditions according to institutional guidelines and using animal study proposals approved by the Institutional Animal Care and Use Committees at Northwestern University.

### Flow cytometry and cytokine production analysis

Single-cell suspensions of thymocytes and splenocytes were used for staining using specific antibodies against CD3 (catalogue no. 100204), CD4 (catalogue no. 100408), CD8 (catalogue no. 100733), CD25 (catalogue no. 102215), CD44 (catalogue no.103011) and CD62L (catalogue no. 104412), all of which were purchased from Biolegend (San Diego, CA), for 30 min on ice. The stained cells were washed with ice-cold PBS, fixed and analysed by flow cytometry as reported^33^. For intracellular staining, cells were fixed and permeabilized using reagents from Biolegend and stained with fluorescent-labelled antibodies against FoxP3 or each specific cytokine.

### *In vitro* T-cell activation and polarization

Naive CD4 T cells were purified using the anti-CD4 antibody-coated magnetic beads (Life Technology, Grand Island, NY). For proliferation, cells were stained with 5 μM of CFSE at room temperature for 15 min followed by adding fetal calf serum (to a final of 2%) to terminate the reaction. CFSE-stained T cells were cultivated with soluble anti-CD3 (eBiosience, 16-0031-86) plus anti-CD28 (eBiosience 16-0281-86; 5 μg ml^−1^) for 3 days and the cell division was analysed by flow cytometry. For T-cell polarization, CD4^+^ T cells were cultivated under each polarization conditions for 5 days. The polarized T cells were analysed by intracellular staining of IFN-γ (Th1; catalogue no. 506908), IL-4 (Th2; catalogue no. 504105), IL-17 (Th17; catalogue no. 506916) and FoxP3 (Treg; catalogue no. 602107), which were purchased from Biolegend, as described^33^.

### Antigen-specific T-cell immune response in mice

Eight- to 12-week-old WT and Hrd1 cKO mice were immunized with 50 μg NP-KLH in 100 μl of CFA on day 1 followed by a boosted immunization with NP-KLH/IFA on day 8. Sera were collected on day 7 and 14 for the detection of NP-specific antibody production by enzyme-linked immunosorbent assay, as described^20^. Immunized mice were killed on day 14 and the total splenocytes were isolated. Antigen-specific T-cell proliferation was analysed by CFSE (Life Technologies, Boston, MA, catalogue no. C34554) staining, and the production of cytokines by CD4 T cells was determined by intracellular staining and flow cytometry analysis.

### Intracellular cytokine staining

*In vitro* stimulated T-cells under either non-polarization or each indicated polarization condition, or cells from the draining spleens of immunized mice were stimulated with phorbol myristate acetate (10 ng ml^−1^; Sigma-Aldrich, P1585), ionomycin (1 μg ml^−1^; Sigma-Aldrich, I0634) and monensin (10 μg ml^−1^; Affymetrix Inc., catalogue no 00-4505-51) for 4 h, and the intracellular staining was performed using an eBioscience kit (San Diego, CA) following a protocol provided by the manufactures.

### Real-time quantitative PCR with reverse transcription

WT and Hrd1 cKO CD4 T cells were activated with or without anti-CD3 plus anti-CD28 for 4 h. Total RNA was extracted with Trizol reagent according to the manufacturer's instructions (Thermo Fisher Scientific, catalogue no. 15596018). Complementary DNA was synthesized using the qScript cDNA Synthesis kit (Quanta Biosciences, Gaithersburg, MD, catalogue no. 95047-100). iQ5 and SYBRGreen Detection system (Bio-Rad, Hercules, CA) were used for quantitative PCR as described^47^,^48^. Data were normalized to the expression of β-actin in each sample. Primers used to detect the expression of Hrd1, p27^kip1^ (Cell Signalling, catalogue no. 3688S), p53 (Abcam, catalogue no. ab61241) and Skp2 (Cell Signalling, catalogue no. 4313S) transcripts are shown in Supplementary Table 1.

### Co-immunoprecipitation and western blotting

Cells were lysed in cold radio immunoprecipitation assay buffer (Life Technologies). The cell lysate was pre-cleaned with protein-G sepharose (GE Healthcare, catalogue no. 17-0618-02) for 30 min and subjected to immunoprecipitation with each indicated antibodies (1 μg), incubated for 1 h on ice and then 25 μl of protein-G sepharose beads were added for additional 4–6 h. The beads were then washed for four times, boiled with 15 ml of 2 × SDS sample buffer for 5 min and the proteins were separated on 10% SDS–PAGE gels and transferred to polyvinylidene difluoride membranes. The membranes were blocked in 5% fat-free dried milk in Tris-buffered saline with 0.5% Tween 20 (TBST) for 2 h. The membranes were then incubated in appropriate primary antibodies overnight at 4 °C. Membranes were washed in TBST and then incubated in horseradish peroxidase (HRP)-conjugated secondary antibodies (EMD Millipore Corp, goat anti-rabbit IgG antibody, HRP conjugate, catalogue no. 12–348; goat anti-mouse IgG antibody, HRP conjugate, catalogue no. 12–349). Membranes were washed in TBST, and the signals were visualized using enhanced chemiluminescence substrate (Thermo Scientific, Waltham, MA) and quantified using the Bio-Rad Image software. Representative uncropped blots are shown in Supplementary Fig. 9.

### EAE induction and disease characterization

Eight- to 10-week-old WT and Hrd1 cKO mice in the C57/BL6 genetic background were immunized with MOG_35–55_ peptide (emulsified with CFA (200 μg per mouse)). Mice were also given pertussis toxin (200 ng per mouse) on days 0 and 2 via tail vein injection. All mice were weighed and examined daily for symptoms and assigned scores on a scale of 0–5 as follows: 0, no overt signs of disease; 1, limp tail; 2, limp tail and partial hindlimb paralysis; 3, complete hindlimb paralysis; 4, complete hindlimb and partial forelimb paralysis; and 5, moribund state or death. For the adoptive transfer, 5 × 10^6^ WT or Hrd1 cKO CD4 T cells were adoptively transferred into T-cell-null (TCRβ/δ DKO mice by intravenous injection. Two days after the transfer, EAE induction was performed and scored.

### Isolation and characterization of human CD4 T cells

About 10 ml fresh blood was collected from each MS patient or healthy donor and centrifuged. After lysis of the red blood cells, total white blood cells were subjected to CD4 T-cell isolation using a negative selection isolation kit (Miltenyi Biotech, San Diego, CA) following the instruction. Purified CD4 T cells were utilized for the following experiments: for RNA purification and real-time PCR analysis, isolated cells were suspended in Trizol reagent and total RNA was isolated according to the manufacturer's instructions (Invitrogen). For the shRNA knockdown experiment, CD4 T cells were infected with lentivirurs carrying Hrd1-specific or control shRNA. Green fluorescent protein-positive cells were either gated or sorted for analysis by intracellular staining or western blotting. The study has been approved by the Institutional Review Board.

### Statistic analysis

All data are indicated as mean±s.d. The unpaired, nonparametric Student's *t*-test (Mann–Whitney test) was used for the statistic analysis. *P*<0.05 was considered as the significant difference.

### Data availability

The data that support the findings of this study are available from the corresponding author on request.

## Additional information

**How to cite this article:** Xu, Y. *et al*. The ER membrane-anchored ubiquitin ligase Hrd1 is a positive regulator of T-cell immunity. *Nat. Commun.* 7:12073 doi: 10.1038/ncomms12073 (2016).

## Supplementary Material

Supplementary Information Supplementary Figures 1-9 and Supplementary Table 1.
