# Supplementary material for: The ER membrane-anchored ubiquitin ligase Hrd1 is a positive regulator of T-cell immunity
Source: Nat Commun. 2016 Jul 15;7:12073. doi: 10.1038/ncomms12073 (PMC4947160; doi:10.1038/ncomms12073)
Supplement: Supplementary Information — Supplementary Figures 1-9 and Supplementary Table 1. [file ncomms12073-s1.pdf]

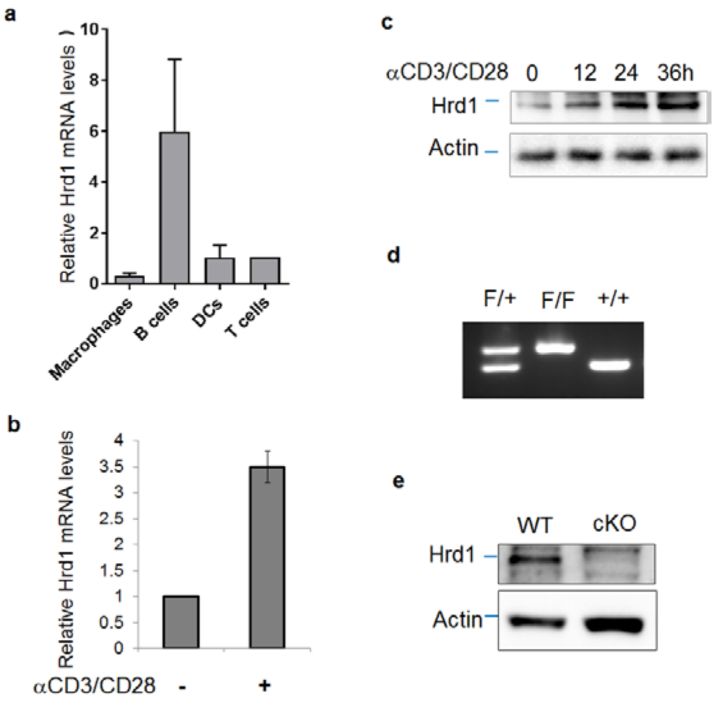

Supplementary Fig. 1. Analysis of Hrd1 expression and genotyping of Hrd1 targeted mice. **(a-c)** Primary immune cells isolated from the spleen of wild type mice. (a) Hrd1 mRNA expression levels in macrophages, B cells, dendritic cells and T cells were analyzed by real-time PCR. Error bars represent data from three independent experiments. (b) CD4 T cells were stimulated with anti-CD3 plus anti-CD28 (2 μg/ml each) for 6 hours, Hrd1 mRNA levels were determined by real-time PCR. (c) CD4 T cells were stimulated with anti-CD3 plus anti-CD28 (2 μg/ml each) for each indicated time. Hrd1 protein expression levels were analyzed by western blotting using anti-Hrd1 antibody (top panel). β-Actin was analyzed as a loading control (bottom panel). **(d)** Genomic DNA purified from the tail snips of wild-type (+/+), heterozygous (F/+) and homozygous (F/F) Hrd1 floxed mice were used for PCR analysis. **(e)** CD4 T cells from WT and Hrd1 conditional knockout (cKO) mice. The protein expression levels of Hrd1 (top panel) and β-Actin (bottom panel) were determined by western blotting.

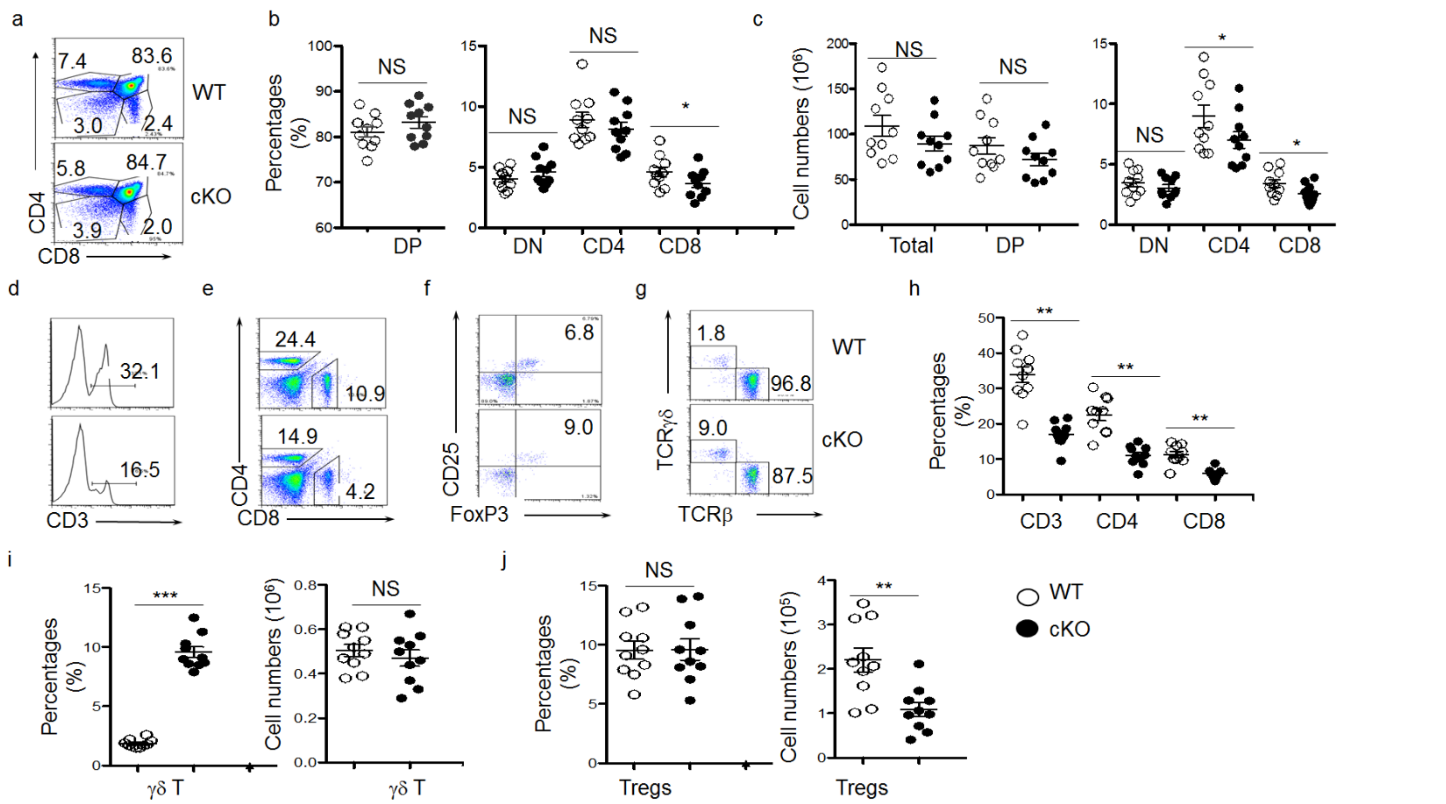

Supplementary Fig. 2. Cellularity analysis of Hrd1-null mice. Single cell suspensions of thymus **(a-c)** and spleen **(d-j)** from wild type (WT) and Hrd1 cKO mice were stained with fluorescent-labeled antibodies against each of the indicated surface markers or FoxP3 and analyzed by flow cytometry. Representative images (a, d-g) are shown, and both the percentages and absolute numbers of T cells from 10 pairs of mice are indicated (b, c, h-j). Mann-Whitney test was used for the statistical analysis. NS, no significant difference; * $p<0.05$, ** $p<0.01$, and *** $p<0.001$.

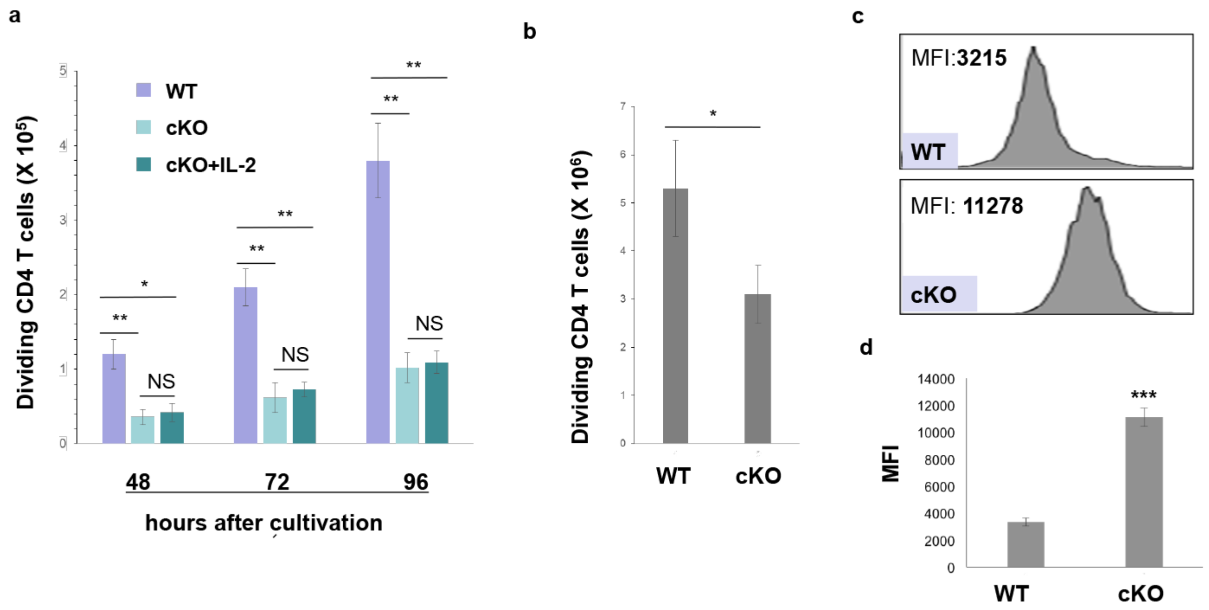

Supplementary Fig. 3. Proliferation of Hrd1-null T cells. (a & b) The absolute numbers of the dividing cells based on the percentage of dividing cells in Fig. 1a & b upon in vitro stimulation (a) or in vivo indicated in Fig. 1f are shown (b). **(c & d)** Naïve $CD8^+$ T cells were isolated from WT and Hrd1 cKO mice and stained with CFSE. Cells were then cultivated with anti-CD3 plus anti-CD28 for three days. The proliferation was determined by flow cytometry analysis of CFSE dilution. The representative images are shown (c) and the mean fluorescent intensity from three independent experiments (d). Mann-Whitney test was used for the statistic analysis. * p<0.05; ** p<0.01 and *** *p<0.001*.

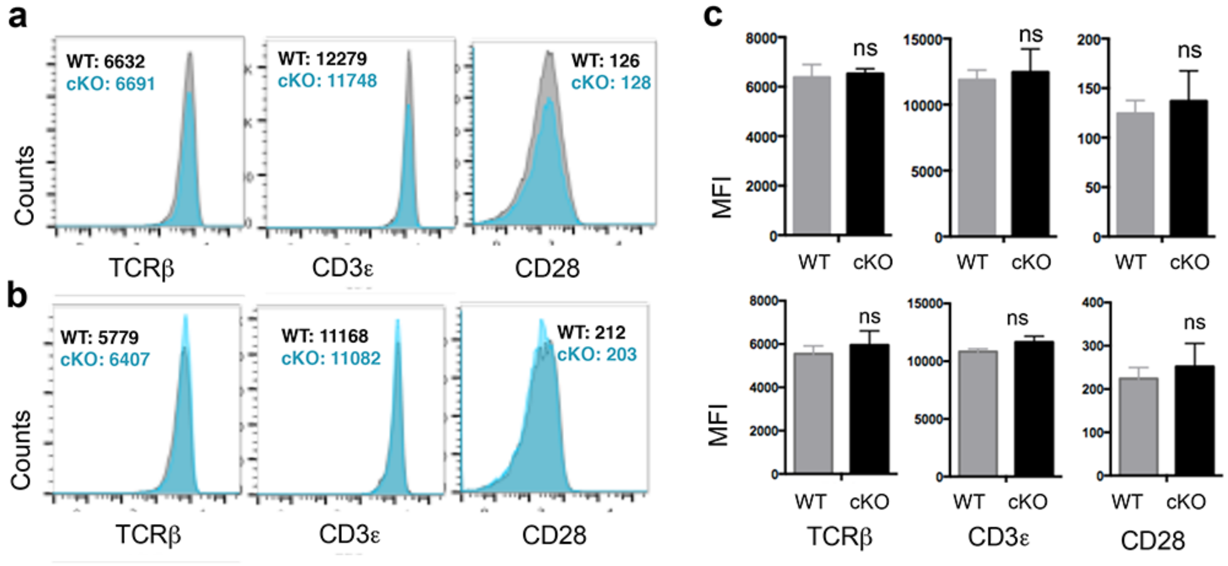

Supplementary Fig. 4. TCR and CD28 expression on Hrd1-null T cells. Single cell suspension was subjected to cell surface staining with fluorescence-conjugated antibodies specific to TCRβ chain, CD3ε and CD28. The expression levels of TCRβ, CD3ε and CD28 on gated $CD4^+$ in spleen (a) and lymph nodes (b) were analyzed. Representative images are shown (a & b). Error bars represent data from 6 pairs of mice (c). Mann-Whitney test was used for the statistic analysis. NS, No significant differences.

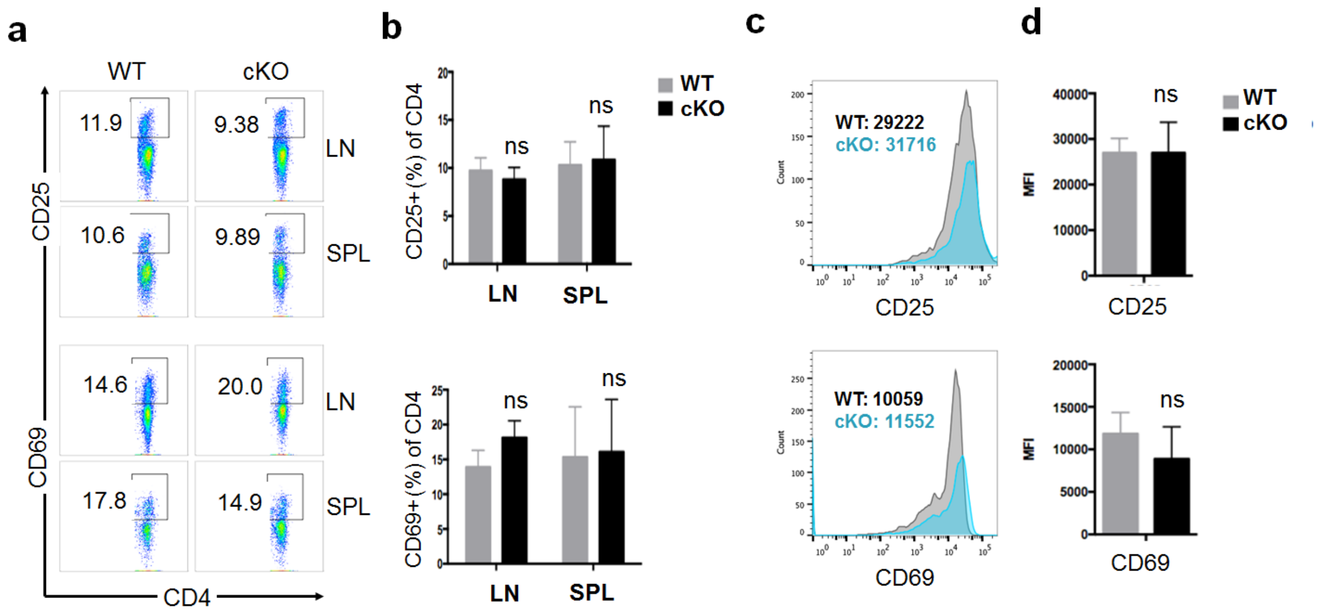

Supplementary Fig. 5. CD25 and CD69 expression on Hrd1-null T cells. (a & b) Single cell suspension was subjected to cell surface staining with fluorescence-conjugated antibodies specific to CD4, Cd25 and CD69. The expression levels of CD25 and CD69 on gated CD4 T cells were analyzed. **(c & d)** Purified $CD4^+$ T cells were stimulated with anti-CD3 plus anti-CD28 (1 µg/ml each) for 24 hours. The expression levels of CD25 and CD69 were determined by flow cytometry. Representative images are shown (a & c). Error bars represent data from 6 pairs of mice (b & d). Mann-Whitney test was used for the statistic analysis. NS, No significant differences.

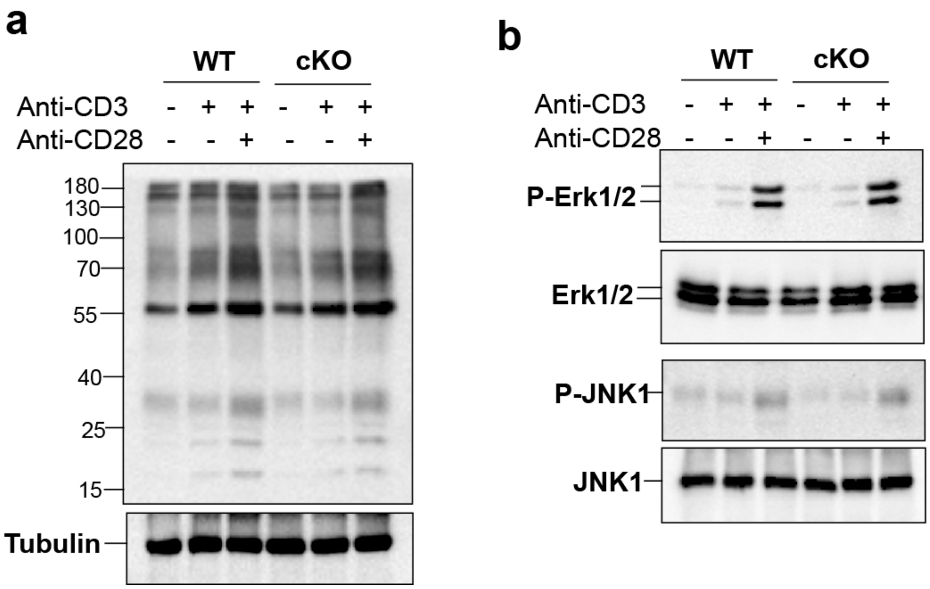

Supplementary Fig. 6. Analysis of the TCR/CD28 signaling in CD4 T cells. Naïve CD4$^+$ T cells were isolated from WT and Hrd1 cKO mice and cultivated with anti-CD3 plus anti-CD28 (1 μg/ml each) for 30 mins. Cell lysates from the stimulated cells were subjected to SDS-PAGE. **(a)** The levels of overall tyrosine phosphorylation were determined by western blotting analysis with anti-phospho-tyrosine antibodies (4G10) (top panel) with Tubulin as a loading control (bottom panel). **(b)** MAPK activation was determined by antibodies specific to phosphorylated Erk1/2 (top panel) and JNK1 (3rd panel). The total protein expression levels of Erk1/2 (2nd panel) and JNK1 (bottom panel) were determined as controls.

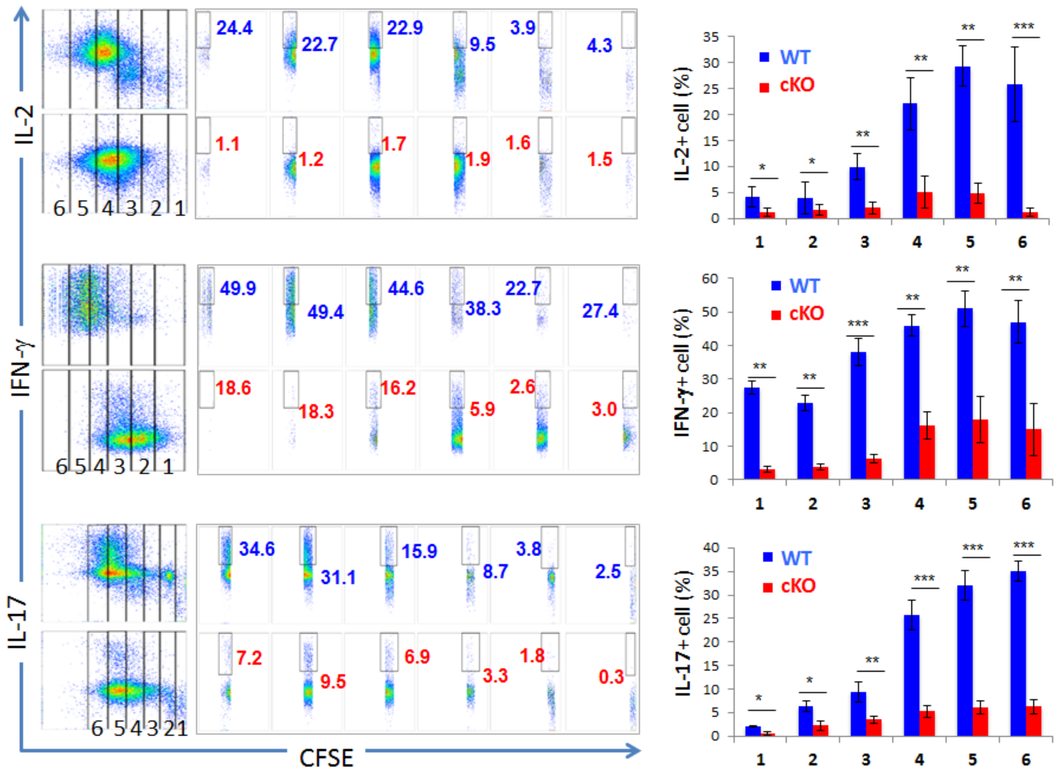

Supplementary Fig. 7. Analysis of the cell cycle-dependent cytokine production of Hrd1-null T cells. Naïve CD4+ T cells were isolated from WT and Hrd1 cKO mice and cultivated under non-polarization (Th0) or Th1 or Th17 skewing conditions for 5 days. The production of IL-2 in Th0, IFN-γ in Th1 and IL-17A in TH17 cells were determined by intracellular staining. The cytokine productions against cell cycle progression (CFSE dilution) were analyzed by flow cytometry. Representative data from 5 independent experiments are shown. Mann-Whitney test was used for the statistic analysis. * $p<0.05$, ** $p<0.01$ and *** $p<0.005$.

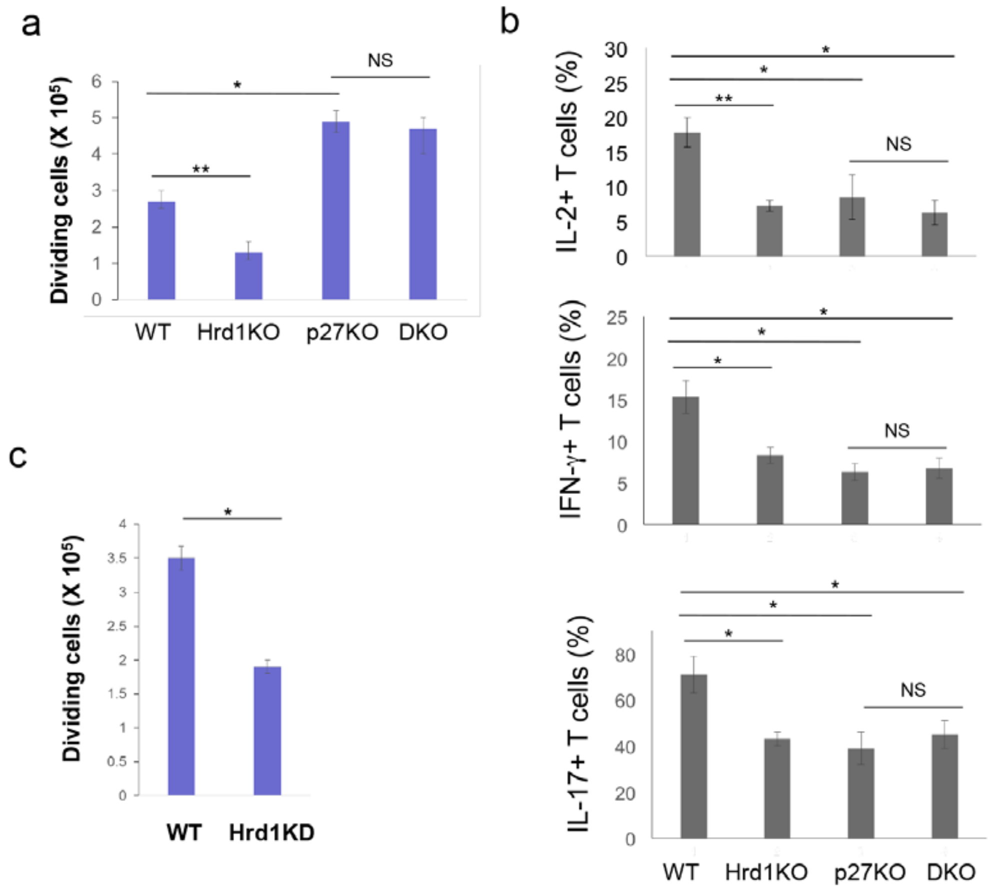

Supplementary Fig. 8. Analysis of the cytokine production by Hrd1/p27^{kip1} double KO T cells and in human T cells. **(a)** The absolute numbers of the dividing cells based on the percentage of dividing cells in Fig. 5f upon in vitro stimulation are shown. **(b)** Naïve CD4 T cells from WT, Hrd1 cKO, p27^{kip1} KO, and dKO mice were cultivated under Th1, Th17, or Treg polarization conditions for 5 days. The production of IFN-γ for Th1 and IL-17A for Th17 and the expression of FoxP3 for Treg were determined by flow cytometry. **(c)** The absolute numbers of the dividing Hrd1 knockdown human CD4 cells based on the percentage of dividing cells in Fig. 7h upon in vitro stimulation are shown. Representative images are shown in Fig. 5k. Data (mean ± SD) from 5 independent experiments are indicated. Mann-Whitney test was used for the statistic analysis.

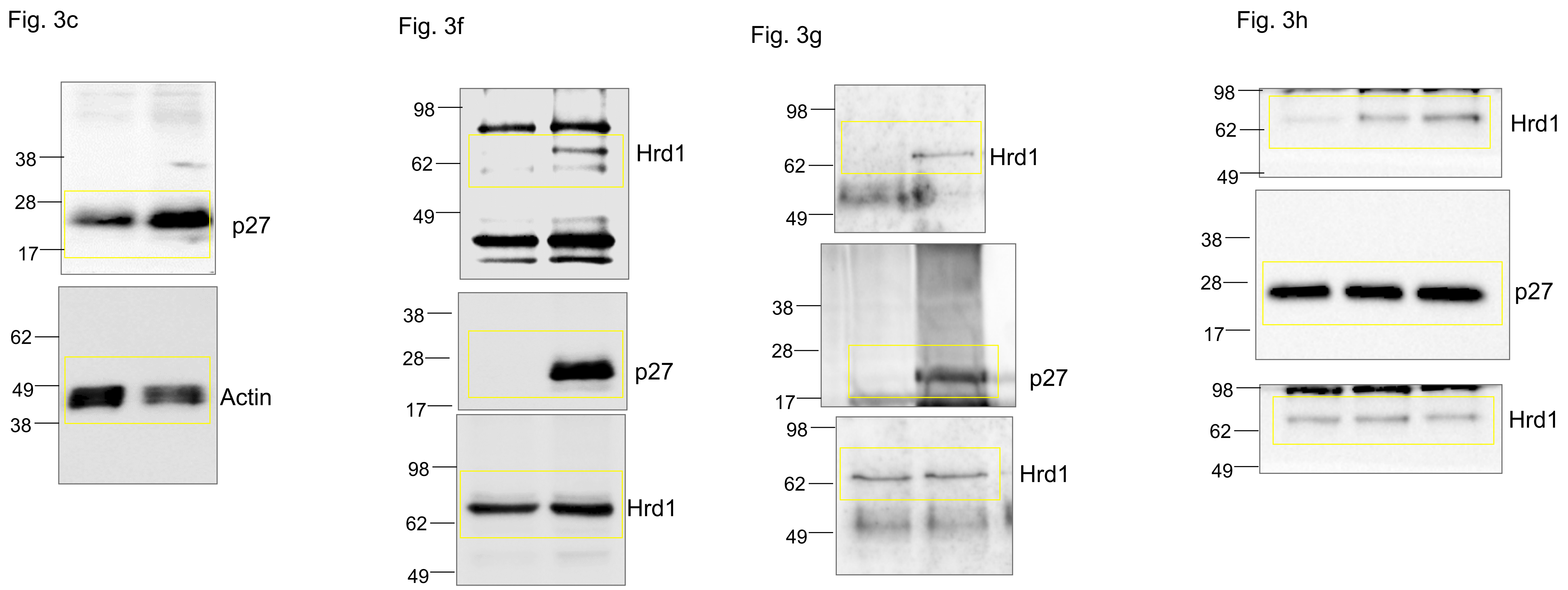

Supplementary Fig. 9a. Uncropped blots for Fig. 3. Note that yellow dashed represents the cropped image.

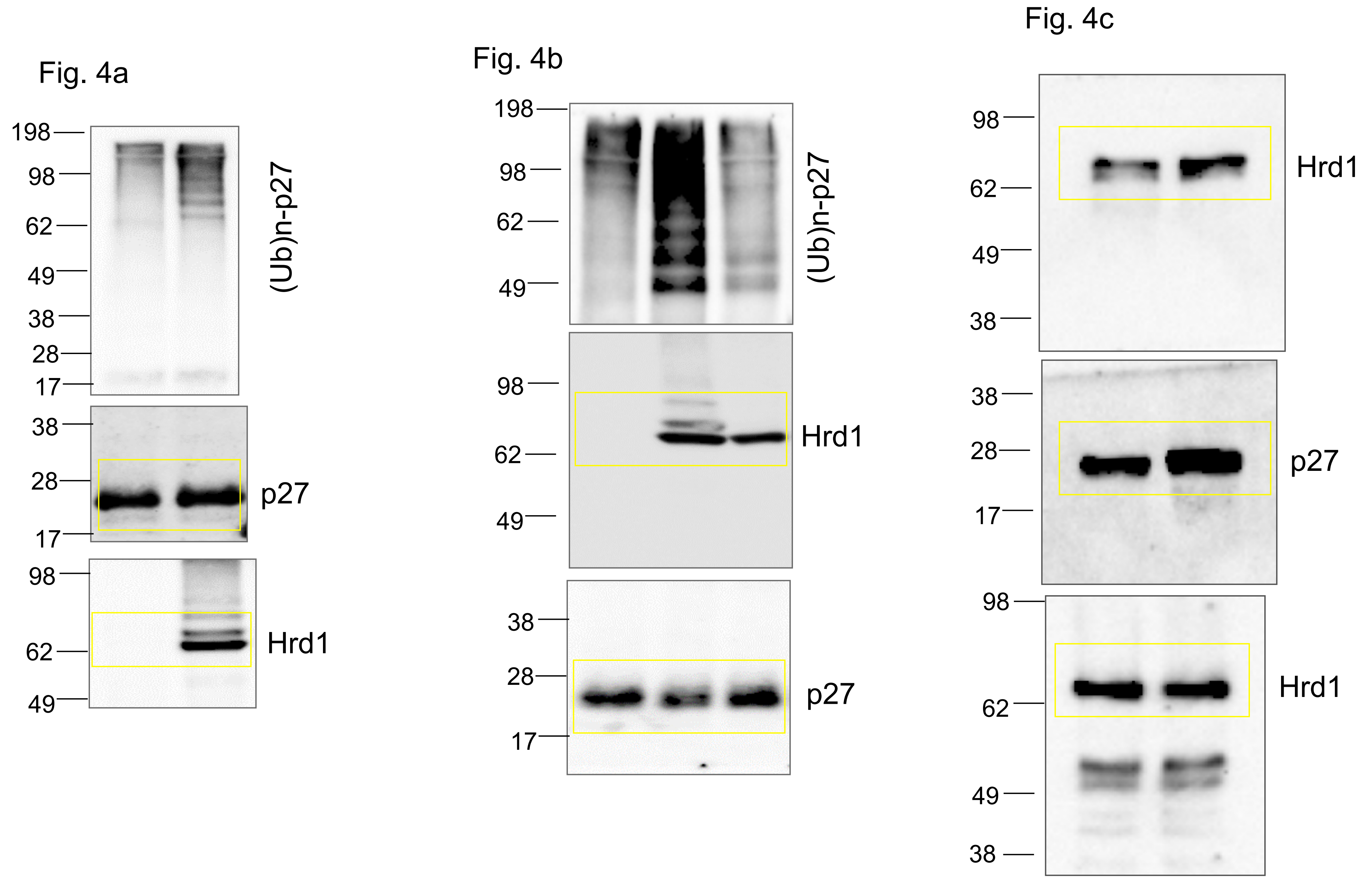

Supplementary Fig. 9b. Uncropped blots for Fig. 4a-c. Note that yellow dashed represents the cropped image.

Fig. 4d

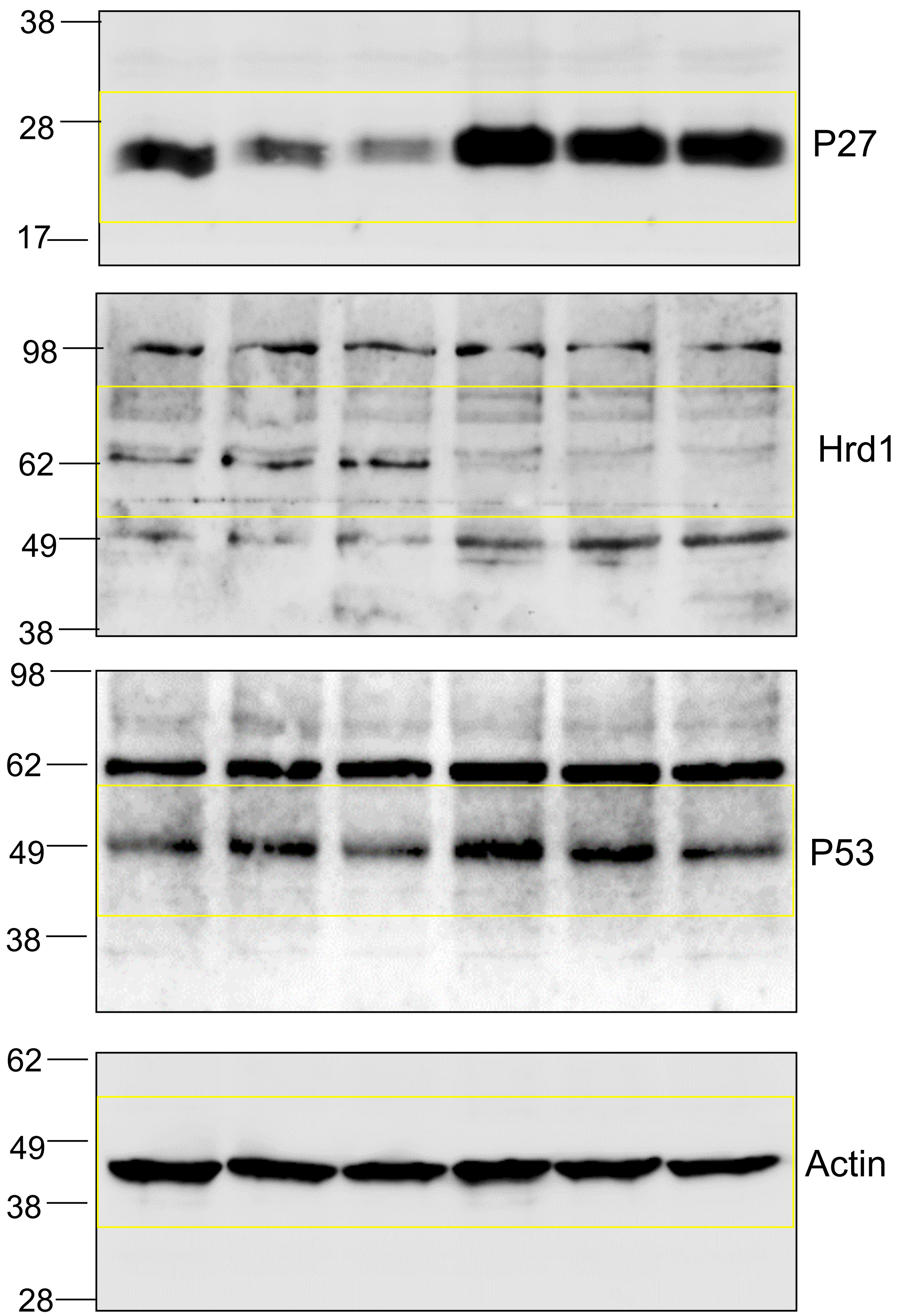

Fig. 4f

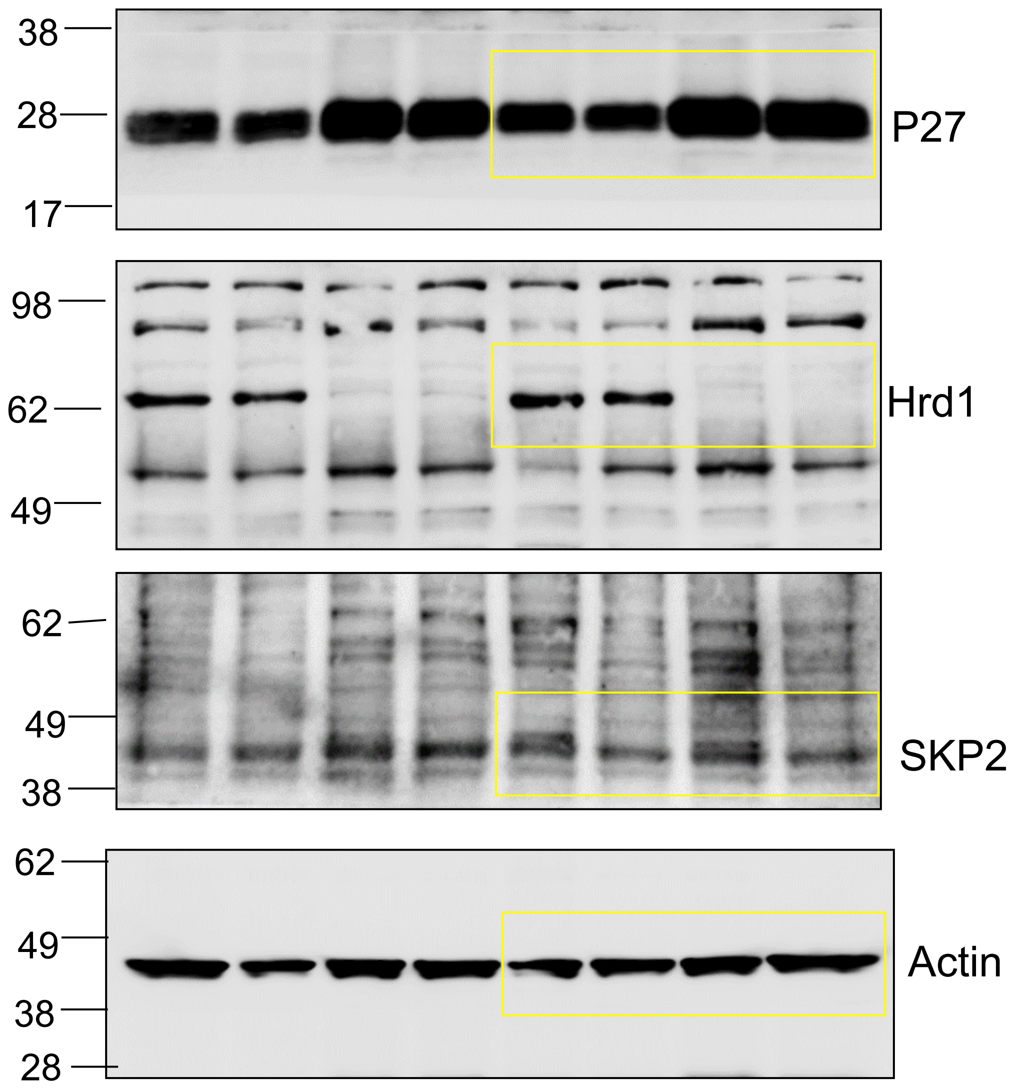

Supplementary Fig. 9c. Uncropped blots for Fig. 4d & f. Note that yellow dashed represents the cropped image.

Fig. 7b

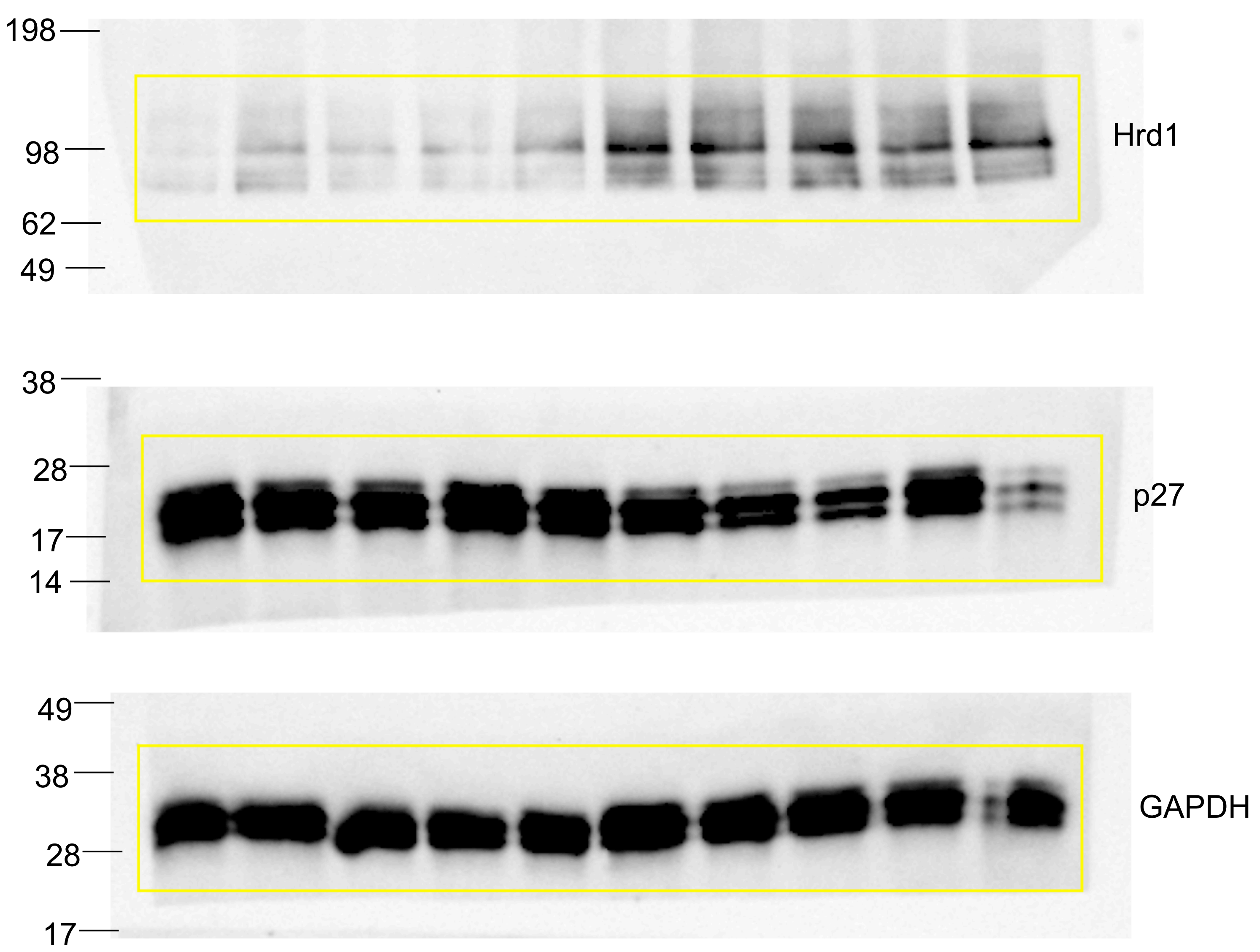

Fig. 7e

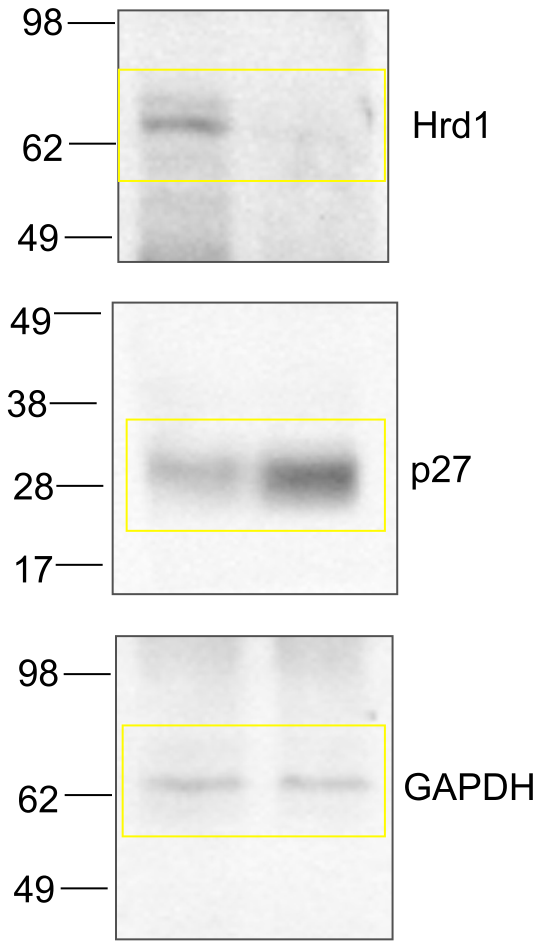

Fig. 7f

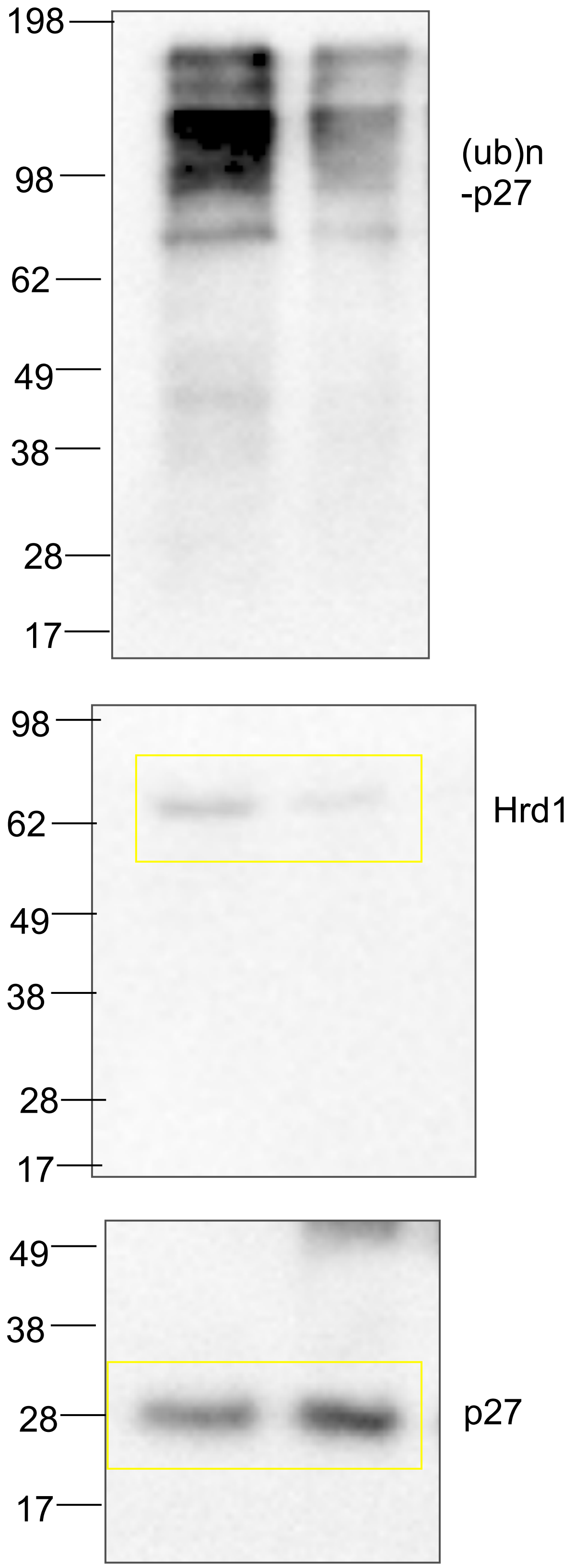

Supplementary Fig. 9d. Uncropped blots for Fig. 7. Note that yellow dashed represents the cropped image.

Suppl. Fig. 1c

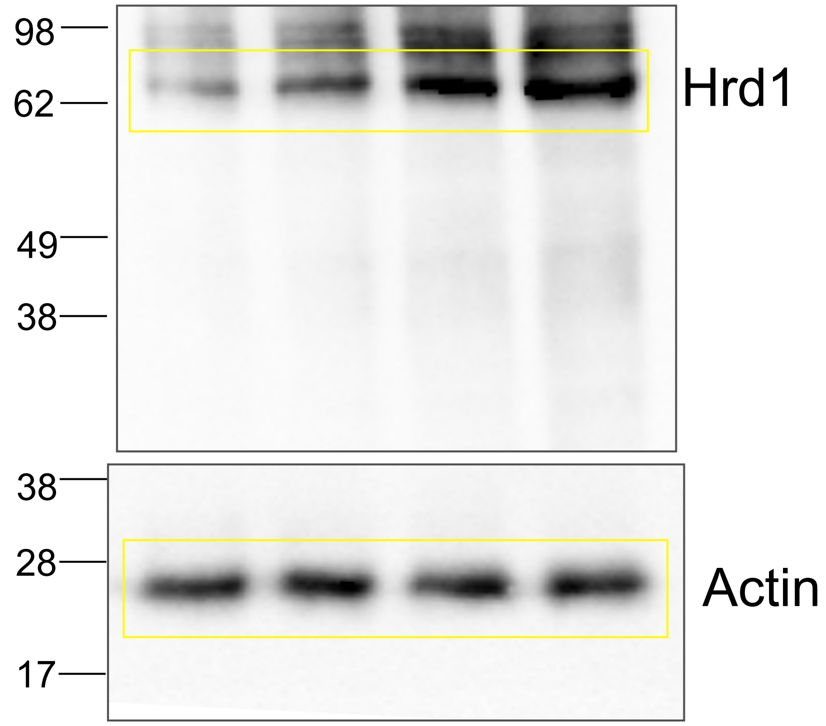

Suppl. Fig. 1b

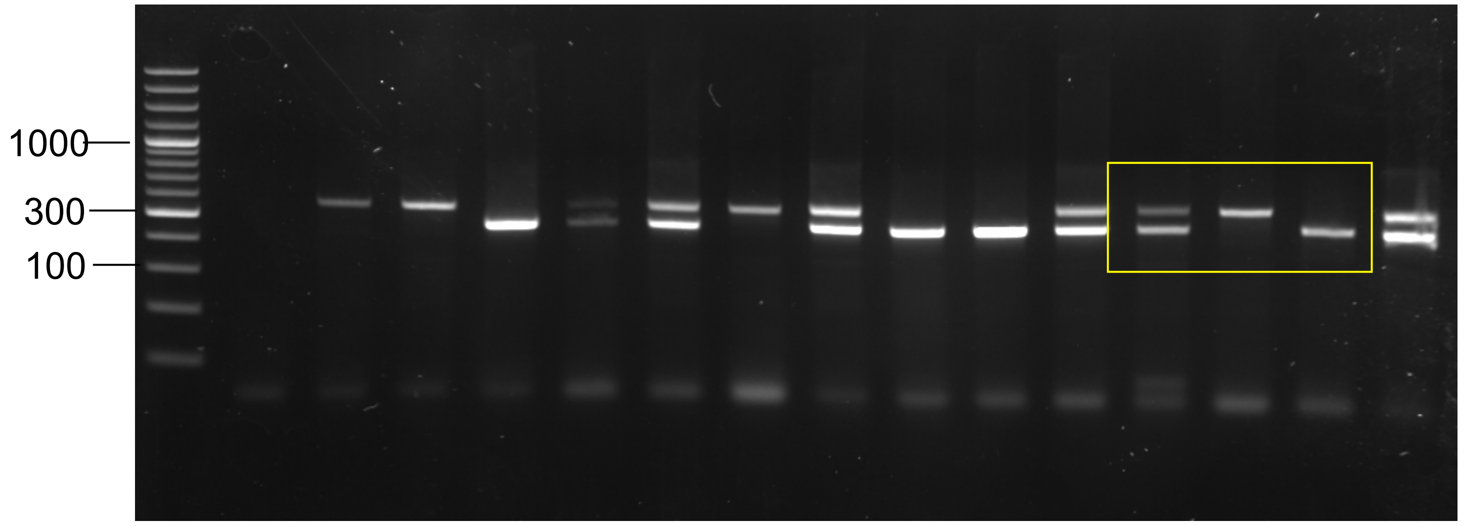

Suppl. Fig. 1e

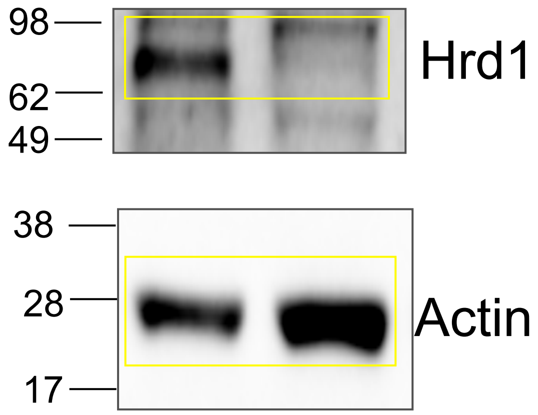

Supplementary Fig. 9e. Uncropped blots for supplemental Fig. 1. Note that yellow dashed represents the cropped image.

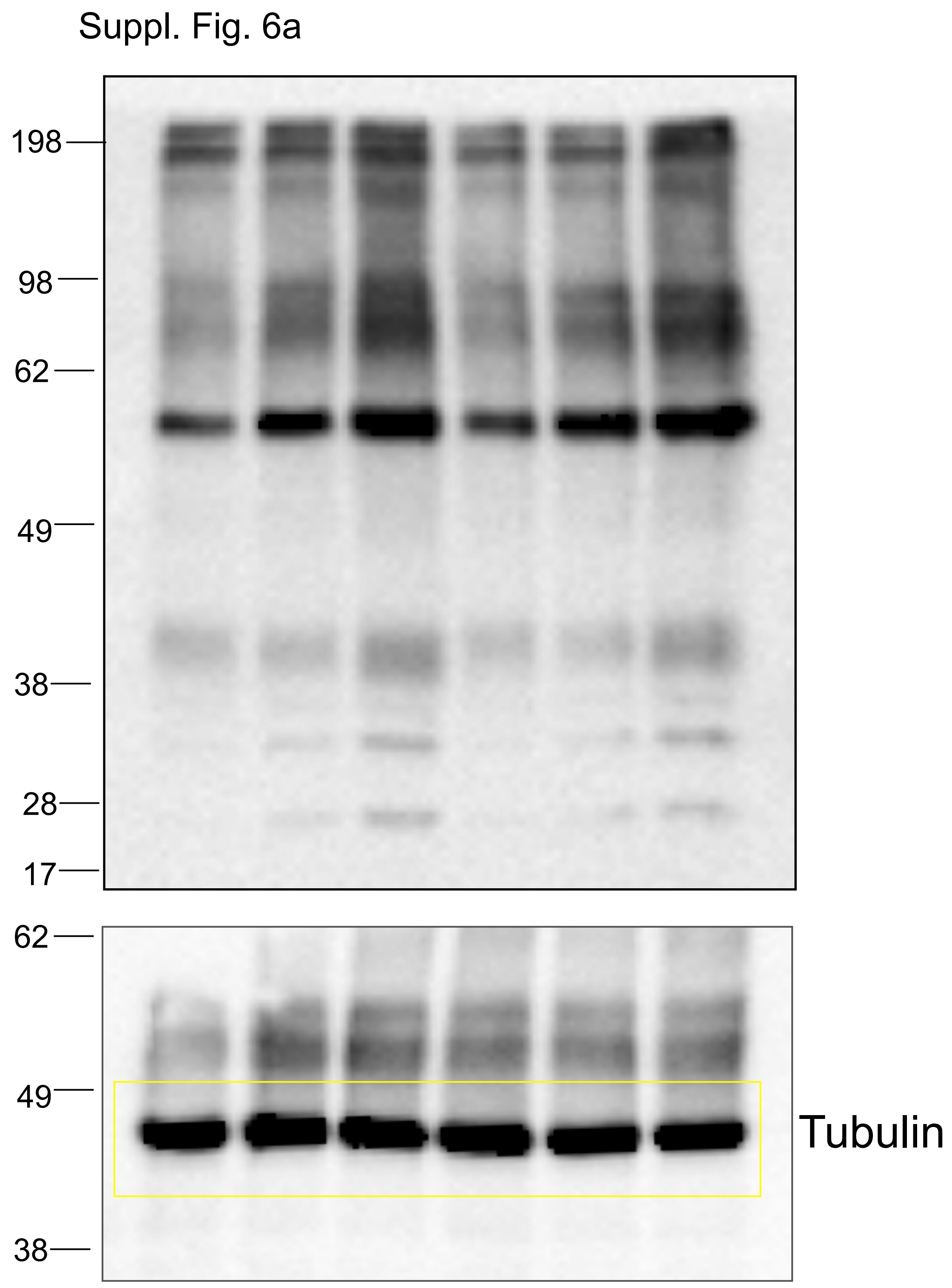

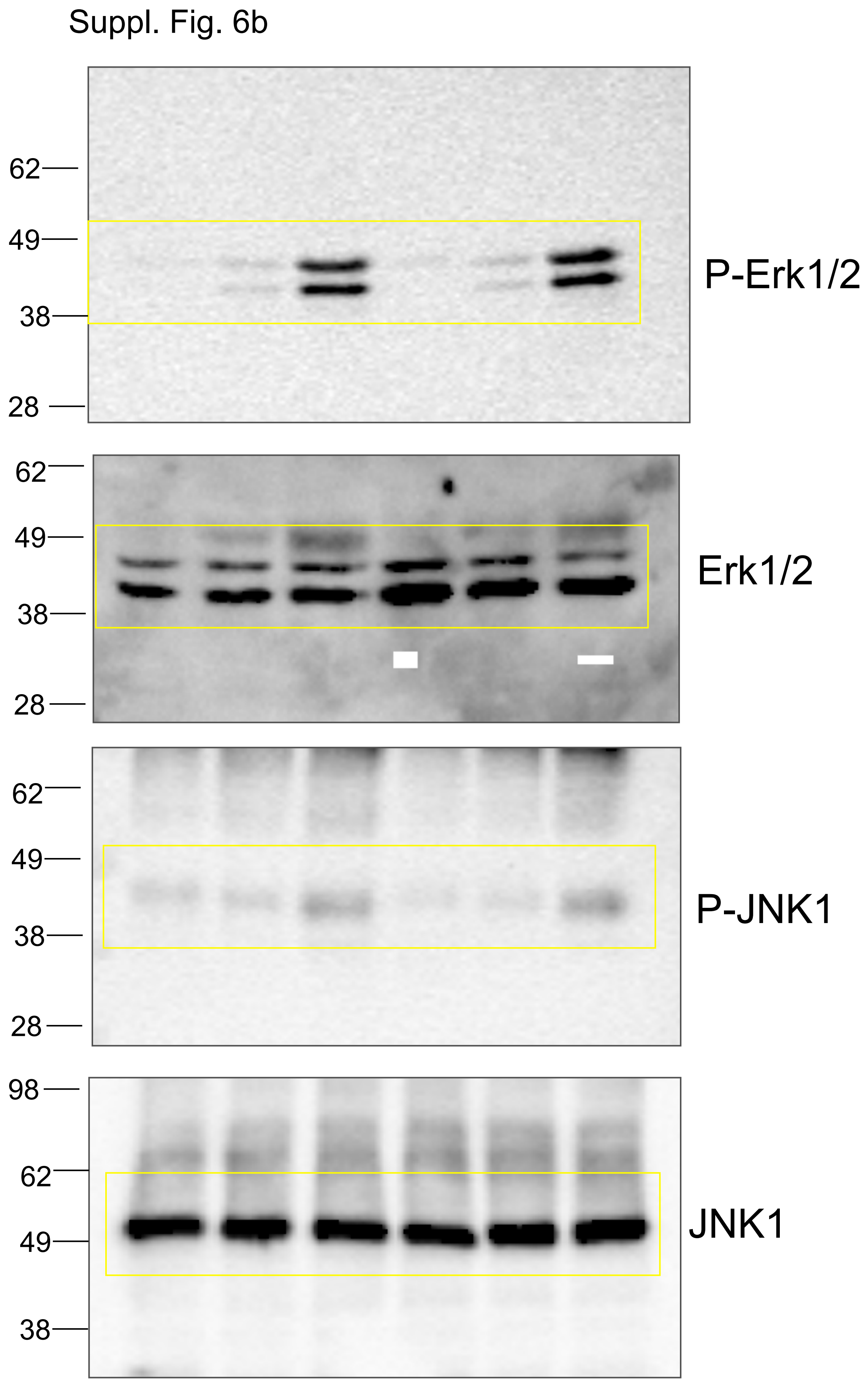

Supplementary Fig. 9f. Uncropped blots for supplemental Fig. 6. Note that yellow dashed represents the cropped image.

Supplemental Table 1. Primers used for qPCR analysis in this study.

Primer set	Sense primer 5' → 3'	Antisense primer 5' → 3'
Xbp1	AAGAACACGCTTGGGAATGG	CTGCACCTGCTGCGGAC
Xbp1s	AGTTAAGAACACGCTTGGGAAT	AAGATGTTCTGGGGAGGTGAC
CHOP	GTCCCTAGCTTGGCTGACAGA	TGGAGAGCGAGGGCTTTG
BiP	TCATCGGACGCACTTGGA	CAACCACCTTGAATGGCAAGA
Erdj3	CCCCAGTGTCAAACTGTACCAG	AGCGTTTCCAATTTTCCATAAATT
puma	TGGGGTCTGCCCAGGCATGT	CGCTCCCTGGGGCCACAAAT
P21	GGAGACTCTCAGGGTCGAAA	GGATTAGGGCTTCCTCTTGG
Hrd1	AGTTTTCGGCCTGTCAGATGG	CGGTGAAGGCCAAACAAGTC
